# Characterization of *Silybum marianum* and *Silybum eburneum* seed oils: Phytochemical profiles and antioxidant properties supporting important nutritional interests

**DOI:** 10.1371/journal.pone.0304021

**Published:** 2024-06-14

**Authors:** Samah Maaloul, Imen Ghzaiel, Maher Mahmoudi, Hédi Mighri, Vivien Pires, Anne Vejux, Lucie Martine, Jean-Paul Pais de Barros, Emmanuelle Prost-Camus, Fayçal Boughalleb, Gérard Lizard, Raoudha Abdellaoui

**Affiliations:** 1 Laboratory of Rangeland Ecosystems and Valorisation of Spontaneous Plants and Associated Microorganisms (LR16IRA03), Arid Regions Institute, University of Gabes, Medenine, Tunisia; 2 Team ‘Biochemistry of the Peroxisome, Inflammation and Lipid Metabolism’ (EA7270) University of Bourgogne/Inserm, Dijon, France; 3 University Clermont Auvergne, Clermont Auvergne INP, CNRS, Institut Pascal, Clermont-Ferrand, France; 4 Laboratory of Plant, Soil and Environement Interactions (LR21ES01)-University of Tunis El-Manar, Faculty of Sciences of Tunis, El-Manar, Tunis, Tunisia; 5 Laboratory of Functional Physiology and Valorization of Bio-Ressources, Higher Institute of Biotechnology of Beja (LR23ES08), University of Jendouba, Jendouba, Tunisia; 6 Centre des Sciences du Goût et de l’Alimentation, CNRS, INRAE, Institut Agro, Université de Bourgogne, Dijon, France; 7 INRAE, University of Bourgogne, Dijon, France; 8 Lipidomic Analytical Platform (LAP), University of Bourgogne, Dijon, France; 9 Laboratory LARA-Spiral, Couternon, France; University of Kotli, PAKISTAN

## Abstract

Milk thistle seed oil is still not a well-known edible oil. *Silybum marianum* (milk thistle), is present in several countries and is the only known representative of the genus *Silybum*. However, *Silybum eburneum*, which is an endemic plant in Spain, Kenya, Morocco, Algeria, and Tunisia, is considered a marginalized species. The present work is the first report that gives information on the lipid and phenolic profiles of Tunisian *S*. *eburneum* seed oil compared to those of Tunisian *S*. *marianum* seed oil. In addition, the antioxidant properties of these oils were determined with DPPH, FRAP, and KRL assays, and their ability to prevent oxidative stress was determined on human monocytic THP-1 cells. These oils are characterized by high amounts of unsaturated fatty acids; linoleic acid and oleic acid are the most abundant. Campesterol, sitosterol, stigmasterol, and β-amyrin were the major phytosterols identified. α-tocopherol was the predominant tocopherol found. These oils also contain significant amounts of phenolic compounds. The diversity and richness of *Silybum marianum* and *Silybum eburneum* seed oils in unsaturated fatty acids, phenolic compounds, and tocopherols are associated with high antioxidant activities revealed by the DPPH, FRAP, and KRL assays. In addition, on THP-1 cells, these oils powerfully reduced the oxidative stress induced by 7-ketocholesterol and 7β-hydroxycholesterol, two strongly pro-oxidant oxysterols often present at increased levels in patients with age-related diseases. *Silybum marianum* and *Silybum eburneum* seed oils are therefore important sources of bioactive molecules with nutritional interest that prevent age-related diseases, the frequency of which is increasing in all countries due to the length of life expectancy.

## 1. Introduction

A balanced diet is a crucial factor in determining human health and preventing age-related diseases including cardiovascular, ocular, and neurodegenerative diseases, as well as inflammatory diseases and diabetes type 2. Currently, many functional foods contain bioactive compounds derived from plant seed oils, such as tocopherols, carotenoids, phytosterols, polyphenols as well as monounsaturated, and polyunsaturated fatty acids, in the form of triglycerides [1–3]. Recently, there has been growing interest on oils derived from non-traditional seeds, such as pomegranate seeds, grape seeds, pumpkin seeds, and milk thistle seeds [4–8].

The Milk thistle is a plant present in several countries, including Mediterranean countries, Southern Russia, Anatolia, Southern Australia, North and South America, and some parts of Asia [9–12]. The genus *Silybum*, which belongs to the *Asteraceae* family, comprises two species: *Silybum marianum* (L) Gaerth and *Silybum eburneum* Coss. and Durieu. These two wild spinescent herbaceous plants are annual and biannual, and they frequently grow on marginal land, grassy banks and river flats [12, 13]. *S*. *marianum* is the only known representative of this genus. In contrast, *S*. *eburneum* is considered a marginalized species and is present only in Spain, Kenya, Morocco, Algeria, and Tunisia.

*S*. *marianum*, also known as milk thistle, is commonly used for medicinal and culinary purposes [14]. It has been extensively used to treat a variety of diseases [15]. The seeds of milk thistles contain a mixture of flavonolignans, commonly referred to as silymarin, which represents its primary pharmaceutical component. This active mixture (Sylimarin) includes silybin, isosilybin, silychristin, dihydrosilybin, and silydrianin [16]. In addition to its high silymarin content, *S*. *marianum* is also known for its high oil content which varies from 22 to 31% [9, 10, 17–20]. Milk thistle seed oil contains a high concentration of unsaturated fatty acids, particularly linoleic acid (C18:2 n-6) (omega 6) and oleic acid (C18:1 n-9) [8, 21, 22]. These two unsaturated fatty acids can protect against cardiovascular diseases which are the most frequent age-related diseases in the world [23, 24]. On the other hand, *S*. *marianum* seed oil contains a high level of phytosterols, namely, campesterol, sitosterol, stigmasterol, and a small amount of cholesterol [8, 22, 25, 26]. These different phytosterols have important beneficial effects on human health, including in the elderly [27]. Some of them have anti-inflammatory, anti-diabetic, immunomodulatory, antiparasitic, antifungal, antibacterial, antioxidant, and neuroprotective activities; they are also efficient for cardiovascular disease prevention [28]. Furthermore, a high level of tocopherols, particularly α-tocopherol, was detected in milk thistle seed oil [7, 8, 29].

According to several pharmacological studies, the compounds identified in *S*. *marianum* seed oil, have hepatoprotective, anti-diabetes, anti-inflammatory, analgesic, antioxidant, and antitumor properties [16, 17, 30–32].

Reactive oxygen species (ROS) are molecules produced by cellular metabolism under normal and pathological conditions. Under normal conditions, ROS are maintained at low levels and play important roles in many essential biological processes, including growth, development and apoptosis. However, high levels of ROS can destroy the structure of biological macromolecules, such as lipids (lipid peroxidation of unsaturated fatty acids and cholesterol), proteins (protein carbonylation), and DNA (formation of 8-oxoguanine) [33–35]. Therefore, oxidative stress, which leads to an overproduction of ROS and reactive nitrogen species (RNS), is a significant factor in the development of aging, chronic and degenerative diseases (arthritis, cardiovascular, and neurodegenerative diseases), and cancer [36, 37]. On the other hand, the autoxidation of cholesterol can also cause the formation of oxidized derivatives called oxysterols. The most abundant oxysterols are 7-ketocholesterol (7KC) and 7β-hydroxycholesterol (7β-OH), which can favor aging and age-related diseases [5, 38]. The cytotoxic effects of 7KC and 7β-OH can be reduced by natural molecules (fatty acids, polyphenols, and tocopherols) present in large amounts in the Mediterranean diet and by vegetal oils such as argan and olive oils [22, 39–41]. *S*. *marianum* seed oil showed important cytoprotective effects on some nerve cells treated with cytotoxic oxysterols [8]: the overproduction of ROS in 158 N murine oligodendrocyte cells was significantly reduced by *S*. *marianum* seed oil as well as by α-tocopherol compared to that in cells treated with two oxysterols: 7KC and 24S-hydroxycholesterol (24S-OHC). Similarly, a significant increase in intracellular ROS levels was detected in murine C2C12 myoblast cells treated with 7β-OH, while oxidative stress was significantly reduced by *S*. *marianum* seed oil and α-tocopherol [5].

Several research projects are currently focused on enhancing the value of vegetal oils. To our knowledge, no studies have been conducted to investigate the physicochemical profile and antioxidant properties of *S*. *eburneum* seed oil obtained from plants grown in Tunisia. This study aimed to evaluate and compare the profiles of fatty acids, phytosterols, and tocopherols in *S*. *marianum*, *S*. *eburneum*, and *S*. *marianum* commercial seed oils. Additionally, this study aimed to determine the total phenolic, flavonoid and carotenoid contents, polyphenolic profiles, and antioxidant activity of these oils using DPPH, FRAP, and Kit Radicaux Libre (KRL) assays. The impact of these oils on the oxidative stress induced in human monocytic cells (THP-1) cultured in the presence of 7KC and 7β-OHC, which are increased in patients with age-related diseases and are strong inducers of oxidative stress in several cell lines, was also studied [38, 41].

## 2. Materials and methods

### 2.1. Seed material and oil extraction

Mature seeds of *S*. *marianum* and *S*. *eburneum* were collected in May 2020 from two different regions in Tunisia; from Médenine (33°21’21.4"N 10°29’05.6" E) in the Southeast and from Sidi Bouzid (35°04’42"N 9°20’06" E) in the Centre, respectively. After harvesting, the seeds were cleaned, dried at 40°C in dark till constant weight, and finely powdered using a mortar and a pestle. The choice of plant material and collection site was provided by the “Laboratory of Pastoral Ecosystems and Valorisation of Spontaneous Plants and Associated Micro-organisms” at the Institute of Arid Regions (IRA) in Medenine, Tunisia. The botanical identification of the species was carried out by Prof. Dr. Mohamed Tarhouni, a researcher at the Arid Regions Institute-Médenine, Tunisia. As *S*. *marianum* and *S*. *eburneum* are not protected species, no collection permission was required. The mature *Silybum* seeds under examination were deposited in the seed bank of the same laboratory.

The oils were extracted by the Soxhlet reflux method using petroleum ether as an extraction solvent [42]. After 6 hours of extraction and cooling, the extract solvent was removed with a rotary evaporator at 40°C. The samples were maintained at 4°C for further analyses.

The oil yield is calculated using the following expression:

$$Oil\;yield\;(\%)=\frac{M}{m}\times 100$$

where: M is the weight in grams of the extracted oil and m is the weight in grams of the dry matter test sample.

Commercial milk thistle (*S*. *marianum* L. Gaertn) seed oil from the “Compagnie des sens” (Lyon, France) was used as reference oil (Lot: CHMA-0004-C; DDM: 03/2025). It was extracted from milk thistle grown and processed in France; the extraction of organic virgin oil was carried out by the cold pressing method.

### 2.2. Lipid fraction analysis of *S*. *marianum*, *S*. *eburneum*, and *S*. *marianum* commercial seed oils

#### 2.2.1. Fatty acid analysis

Ten milligrams of oil were submitted to fatty acid methylation using 7% boron trifluoride in methanol according to the method reported by Morrison and Smith [43]. Fatty acid methyl esters (FAMEs) were analyzed using a Trace 1310 gas chromatography with flame ionization detector (FID) (Thermo scientific) using a CPSIL-88 column (100 m × 0.25mm internal diameter i. d.,0.20 μm film thickness, Varian, Les Ulis, France). Hydrogen was used as a carrier gas (inlet pressure, 210 kPa). The oven temperature was held at 60°C for 5 min, increased to 165°C at 15°C/min and held for 1 min, then to 225°C at 2°C/min and finally held at 225°C for 17 min. The injector and the detector were maintained at 250°C and 280°C, respectively. FAMEs were identified by comparison with commercial and synthetic standards. The data were computed using the Chromquest software (Thermo Scientific), analyzed in INRAe (Dijon, France) and expressed as a percentage of total fatty acids.

#### 2.2.2. Sterol analysis

*Sterol extraction*. Fifty mg of oil was saponified with 5 mL of methanolic KOH (0.35 N) in a capped flask for 2h at room temperature. Then, 100 μL phosphoric acid, 3 mL of 0.73% NaCl, and 9 mL of chloroform were added and mixed. The resulting solution was centrifuged and the organic fraction was transferred to a flask. The solvent was evaporated to dryness under nitrogen at 40°C. A total of 11.1 μg of cholestanol as an internal standard was added and the sample was derivatized to trimethylsilyl ether (TMS ether) by the addition of 400 μL of N, O-bis (trimethylsilyl) trifluoroacetamide at 60°C for 30 min, and then injected into the gas chromatograph (GC) [22].

*Sterol quantification by gas chromatography-flame ionization detection (GC-FID)*. Samples were analyzed by GC-FID with a Hewlett-Packard HP-4890D chromatograph equipped with a DB5 MS fused-silica capillary column (30m, 0.25 mm i.d. 0.25 μm film thickness). The oven temperature was raised from 50°C to 290°C at a rate of 20°C/min. The flame ionization detector (FID) temperature was held at 290°C. The split ratio was 1:20. Hydrogen was used as the carrier gas at a pressure of 60 kPa. TMS ethers were eluted from the column. The data were processed using EZChom Elite software (Agilent Technologies, massy, France). The areas of sterols were compared with the areas of known quantities of the internal standard (cholesterol). The data were analyzed at INRAe (Dijon, France) and are expressed as mg/kg of oil.

#### 2.2.3 Tocopherol analysis

*S*. *marianum*, *S*. *eburneum*, and *S*. *marianum* commercial seed oils were diluted fivefold with hexane. Around 10 mg of each diluted sample was mixed with 200 μL of NaCl (9 g/L), 200 μL of Tocol and 500 μL of hexane. The mixture was centrifuged at 10000 g for 5 min at 4°C. Then, 300 μL of the supernatant was collected in a new tube and evaporated to dryness with a nitrogen stream. The dried extract was suspended with 100 μL of methanolic BHT and further centrifuged at 10000 g for 5 min at 4°C. The supernatant was finally transferred to an injection vial. 2 μL of extract was injected with an 1100 autosampler into a Poroshell 120 EC-C18 column (3×50 mm, 2.7 μm) maintained at 35°C. Separation was achieved with a HPLC-Fluo double pump using a linear gradient of methanol (90% up to 100% in 5 min, and maintained at 100% for 3 min). Detection was performed with a Fluorescence Light Detector (Agilent Technologies, Carven Arms, England) at λ _Exmax_ = 292 nm and λ _Emmax_ = 325 nm. Authentic α, γ, and δ-tocopherol standards (0, 5, 10, 20, 40, 80, 160, 320, and 500 ng) were extracted with the same protocol as the oil sample. Area ratios of α, γ, and δ-tocopherol to tocol were calculated for oil and calibrations standards. A linear calibration curve was used for the calculations. The α, γ, and δ-tocopherol analysis was performed by the lipidomic analytical platform (LAP, Université de Bourgogne, Dijon, France).

### 2.3. Phenolic compounds analysis of *S*. *marianum*, *S*. *eburneum*, and *S*. *marianum* commercial seed oils

#### 2.3.1. Procedure of seed oil preparation

Phenolic extracts of milk thistle seed oils were performed according to the method reported by Ghzaiel *et al*. [44]. One gram of each oil sample was extracted with a mixture of 500 μL of n-hexane and 1 mL of methanol/water (60:40, v/v). After vigorous agitation, for 10 min, the extracts were centrifuged at 1490 g for 15 min. The upper phase was recovered, while the other phase was re-extracted twice with 60% methanol. Finally, the three recovered phases (hydroalcoholic fraction) were homogenized and stored at -20°C for further use.

#### 2.3.2. Total phenolic content (TPC)

The TPC was determined using Folin-Ciocalteau’s assay reported by Dewanto *et al*. [45]. In brief, 125 μL of each hydroalcoholic fraction was mixed with 500 μL distilled water and 125 μL of Folin-Ciocalteu reactive solution. After 5 min, 1250 mL of carbonate sodium (Na_2_CO_3_)/ water solution (7:93, v/v) was added. Then, the mixture was adjusted to a final volume of 3 mL with distilled water and thoroughly mixed. After 90 min of incubation at room temperature in the dark, the absorbance was measured at 765 nm. The concentrations are expressed as milligrams of gallic acid equivalents per 100 g of oil (mg GAE/ 100 g oil).

#### 2.3.3. Total flavonoids content (TFC)

The TFC was assayed by the aluminium chloride method reported by Dewanto *et al*. [45]. Each 250 μL of sample was mixed with 75 μL of sodium nitrite (NaNO_2_)/water solution (5:95, v/v). After 5 min, 150 μL of freshly prepared aluminum chloride (AlCl_3_)/ water solution (10:90, v/v) was added. Five minutes later, 500 μL of sodium hydroxide (NaOH) /water solution (4:96, v/v) was added, and the final volume was 3 mL with distilled water. The mixture was thoroughly mixed and incubated for 15 min in the dark. The absorbance was recorded at 510 nm and the result was expressed as milligrams quercetin equivalents per 100 g of oil (mg QRE/100 g oil).

#### 2.3.4. Total carotenoid content (TCC)

The TCC was determined using the method of Dhibi *et al*. [46]. In a glass tube, 50 mg/ oil sample was mixed with 1 mL of petroleum ether. After robust agitation, the absorbance was measured at 440 and 480 nm. The result was expressed using the following formula:

$$Carotenoids=Amax\;\times \;\frac{10^{5}}{2.65}$$

where: A_max_ is the maximum absorption between 440 and 480 nm.

#### 2.3.5. Polyphenol analysis

The identification of phenolic compounds in oils was carried out by HPLC. One gram/oil sample was solubilized in 6 mL of petroleum ether (40–60%) and then loaded on a silica cartridge (previously packed in 6 mL of petroleum ether (40–60%)). The cartridge was rinsed with 12 mL of petroleum ether (40–60%), then eluted with 8 mL of methanol (80%), then with 8 mL of acetonitrile. The mixture was then evaporated under reduced pressure at 50°C to about 1 mL. The final volume was converted into a clean tube and evaporated under nitrogen at 50°C. The residue was diluted in a mixture of methanol, DMSO, and acetone (3:1:1; v/v/v), then centrifuged (10,000 rpm for 10 min) and filtered through a 0.45 μm nylon membrane. The analysis was performed by high-performance liquid chromatography equipped with UV detector on a licrospher 100 RP-18 column (150 × 4.6 mm i.d., 5 μm particle size, Merck) using an elution gradient and dual detection at 280 and 345 nm, calibrated against quercetin as an external reference standard. Compounds were identified using the library of polyphenol spectra (UV spectra + retention time) available at the Lara-Spiral laboratory (Couternon, France) and expressed in milligrams equivalent of quercetin per 100 g of oil.

### 2.4. Antioxidant activity of *S*. *marianum*, *S*. *eburneum*, and *S*. *marianum* commercial seed oils

#### 2.4.1. KRL test

The total antioxidant potential of *S*. *marianum* and *S*. *eburneum*, as well as *S*. *marianum* commercial seed oils, was assessed using the KRL test (Kit Radicaux Libres, Lara-Spiral, Couternon, France). This test allows a dynamic evaluation of the overall resistance of red blood cells subjected to a radical attack [22, 44, 47]. Diluted control blood samples, with or without the three oils, were oxidized by molecular oxygen in an aqueous suspension using a 2.2’-azobis (2-amidinopopane) dihydrochloride (AAPH) solution. Hemolysis was recorded with a 96-well microplate reader (KRL Reader, Kirial International, Couternon, France) by measuring the turbidimetric optical density decay at 620 nm. The oils’ antioxidant efficiency was presented as mg Trolox equivalent and mg gallic acid equivalent per gram of oil. This test was conducted at the Lara-Spiral laboratory located in Couternon, France.

#### 2.4.2. Determination of total antioxidant activity (TAA)

The TAA of each sample was determined using the phosphor-molybdenum method as described by Prieto *et al*. [48]. A volume of 100 μL of each sample wasmixed with 1 mL of reagent solution (0.6 M sulfuric acid (H_2_SO_4_), 28 mM monosodium phosphate (NaH_2_PO_4_), and 4 mM ammonium molybdate ((NH_4_)_6_Mo_7_O_24_.4H_2_O)). After 90 min of incubation at 95°C, the absorbance was recorded at 700 nm. The result was expressed as milligrams of gallic acid equivalents per 100 g of oil (mg GAE/100 g oil).

#### 2.4.3 Determination of DPPH radical scavenging ability

DPPH radical scavenging activity was tested by the method of Sánchez *et al*. [49]. In addition, 50 μL/sample was complemented with 1950 μL of the methanolic 2,2-diphenyl 1-picrylhdrazyle (DPPH) solution (0.025 g/L). The mixture was vortexed and incubated at room temperature in the dark for 30 min. A negative control was prepared under the same conditions by replacing the specimen with 50 μL of methanol. The absorbance of the DPPH radical inhibition was registered at 517 nm. The percentage of the ability to scavenge the DPPH radical was determined as noted below:

$$\frac{Abs\;control-Abs\;sample\;}{Abs\;control}\times 100$$

where: ‘Abs control’ and ‘Abs sample’ are the control absorbance and the sample absorbance, respectively.

The result was expressed as milligrams of Trolox equivalents per 100 g of oil (mg TRE/ 100 g oil).

#### 2.4.4 Ferric Reducing Antioxidant Power (FRAP)

The FRAP was determined according to Oktay *et al*. [50]. A volume of 200 μL of each hydroalcoholic fraction was mixed with 500 μL of phosphate buffer (0.2 M, pH = 6.6) and 250 μL of potassium ferricyanide (K_3_Fe (CN)_6_)/ water (1:99, v/v). After 20 min of incubation at 50°C, 500 μL of trichloroacetic acid (TCA)/ water (10:90, v/v) was added. The mixture was then centrifuged at 3000 g for 10 min. Then, 500 μL of each supernatant was mixed with 500 μL of distilled water and 100 μL of ferric chloride solution (FeCl_3_)/ water (0.1:99.9, v/v). The absorbance was measured at 700 nm and the result was expressed as milligrams of Trolox equivalents per 100 g of oil (mg TRE/ 100 g oil).https://fr.techdico.com/traduction/anglais-francais/trichloroacetic+acid.html

### 2.5 In vitro study

#### 2.5.1 Cell and cell treatment

Human monocytic THP-1 cells were cultured in Roswell Park Memorial Institute 1640 medium (RPMI 1640) (Gibco) supplemented with 10% decomplemented fetal bovine serum (FBS) (Gibco) and 1% penicillin (100 U/mL) and streptomycin (100 U/mL) (Gibco). The cells were maintained in a humidified atmosphere (5% CO_2_) at 37°C. On average, they should be run every 2 to 4 days with a dilution between 1/3 and 1/10 (the cells resist up to twenty passages and it should be maintained at a cell density less than 1×10^6^ and more than 4×10^5^ cells per mL). We grow THP-1 in 8 mL of medium per T25 flask, 2 mL of medium per well of a 6-well plate, 1 mL per well of a 12-well plate or 500 μL per well of a 24-well plate.

A stock solution of 60 mg/mL in dimethyl sulfoxide (DMSO) of *Silybum* seed oils was prepared and stored in the dark at 4°C. On the other hand, the stock solutions of 7-ketocholesterol (7KC) (Sigma-Aldrich, Saint Quentin-Fallavier, France) and 7β-hydroxycholesterol (7β-OHC) (Sigma-Aldrich) were prepared at 2 mM (800 μg/mL) and stored in the dark at 4°C [51]. Similarly, a α-tocopherol solution (Sigma-Aldrich) was prepared at 80 mM in absolute ethanol, and stored in the dark at 4°C.

For the oxidative stress experiments, THP-1 cells were seeded in 24-well plates (2×10^5^ cells per well). After 24 hours, the THP-1 cells were incubated with 7KC and 7β-OHC (62.5 mM; 25 μg/mL) for 24 hours with or without the oils (*S*. *marianum* (SM), *S*. *eburneum* (SE), and *S*. *marianum* commercial (SMC)) (100μg/mL), or α-tocopherol (400μM) (used as a positive control for cytoprotection).

The same condition is used to prepare the toxicity range of vehicles (DMSO, ethanol), oxysterols (7KC and 7β-OHC) and oils (SM, SE, and SMC) relative to THP-1 cells.

#### 2.5.2 Measurement of cell viability with the Fluorescein Diacetate Assay

Fluorescein diacetate (FDA) is a substrate for cell-permeant esterase that can be used as a viability test indicator according to the method of Jones and Senft [52]. After 24 hours of treatment with various percentages of DMSO (0.041 to 1.33%) and ethanol (0.0325 to 1.04%), and different concentrations of oxysterols (15.625 to 500 μM) and (6.25 to 200 μg/mL) and oils (25 to 800 μg/mL), THP-1 cells were incubated with FDA (50 μM) for 5 min at 37°C. The fluorescence intensity of the fluorescein (λ Excitation: 485 nm; λ Emission: 538 nm) was measured with a Tecan fluorescence microplate reader (Tecan Infinite M200 Pro, Lyon, France) to quantify the living cells. The results were expressed as a percentage of control: $\%\;FDA\;positive\;cells\;(\%\;control)=\frac{Fluorescence\;assay}{Fluorescence\;control}\times 100$

#### 2.5.3 Measurement of Reactive Oxygen Species (ROS) production with dihydroethidium

The superoxide anion (O_2_^•-^) production was measured by flow cytometry after staining with dihydroethidium (DHE) according to the method of Rothe and Valet [53]. DHE is a dye that can freely diffuse across cell membranes. It is rapidly oxidized under the action of ROS to fluorescent ethidium. This latter exhibits an orange/red fluorescence (λ_Ex_ Max = 488 nm; λ_Em_ Max = 575 nm). Briefly, after 24h of treatment, THP-1 cells were stained with 2 μM DHE solution for 15 min at 37°C and then analyzed on a BD LSR ^TM^ II flow cytometer (BD Biosciences, San Jose, CA, USA). The fluorescence signals of the DHE-stained cells were collected through a 580 nm band pass filter. For each sample, 10,000 cells were acquired. The data were analyzed with FlowJo.

### 2.6. Statistical analysis

The statistical analysis of the data was performed using SPSS statistical software version 22 (IBM Corp, Armonk, NY, USA). The data are expressed as means ± standard deviation (SD) of three replicates and were compared with an ANOVA test followed by Duncan’s post-Hoc test. A nonparametric test (Kruskal-Wallis test) was used for γ, α, and total tocopherol content and carotenoid content. For *in vitro* test, the data were analyzed with GraphPad Prism 8.0.1 software and expressed as means ± standard deviation (SD) of three replicates. The data of FDA test was compared with ANOVA test followed by Bonferroni’s test, which allows comparison with the control. On the other hand, the data of DHE test was compared with ANOVA test followed by Tukey test, which allows multiple comparisons and permits assessment of any interaction. The significance level was set at 5% to determine the differences between means. The principal component analysis (PCA) and a heatmap, generated using XLSTAT (2019), present the correlations between the parameters and their relationships with the three oils.

## 3. Results

### 3.1. Oil contents, fatty acids, phytosterols and tocopherols profiles of *S*. *marianum*, *S*. *eburneum*, and *S*. *marianum* commercial seed oils

Seed oils of *S*. *marianum*, *S*. *eburneum*, and *S*. *marianum* commercial were characterized. Our results showed that the oil content in *S*. *marianum* and *S*. *eburneum* seeds was around 27.76 and 28.55%, respectively (Table 1). The fatty acid composition is given in Table 2. Information regarding the data on fatty acids can be found in S1 Table. Twenty-one fatty acids were identified in *S*. *marianum*, *S*. *eburneum*, and *S*. *marianum* commercial seed oil. These oils showed higher amounts of unsaturated fatty acids (UFA) with no significant difference between them (*p* = 0.418). The highest UFA was recorded in *S*. *marianum* seed oil (SMSO) (74.35±5.53%) followed by *S*. *eburneum* (SESO) (69.61±13.59%) and *S*. *marianum* commercial seed oil (SMCSO) (64.28±3.21%). Furthermore, the polyunsaturated fatty acids (PUFA), which varied significantly (*p* = 0.022) between 37.73±1.88% (SMCSO) and 55.83±5.62% (SMSO), was higher than monounsaturated fatty acids (MUFA) which ranged from 14.58±4.18% (SESO) to 26.57±1.33% (SMCSO) with no significant difference (*p* = 0.178). On the other hand, a non-significant difference (*p* = 0.062) was observed in the percentage of saturated fatty acids (SFA) with the highest value in SMCSO (30.03±1.50%). However, SMCSO showed the highest significant contents of stearic acid (C18:0), arachidic acid (C20:0), behenic acid (C22:0), and lignoceric acid (C24:0) compared to *S*. *marianum* and *S*. *eburneum* wild plants. Overall, among the identified fatty acids the major detected ones were linoleic acid (C18:2 n-6), oleic acid (C18:1 n-9), palmitic acid (C16:0), stearic acid (C18:0), arachidic acid (C20:0), and behenic acid (C22:0). It is worth noting that *Silybum* oil is essentially composed by the linoleic acid (C18:2 n-6) that is more represented in wild plants oil than the commercial one. On the other hand, the rate of C18:1 n-9 and C16:0 presented no significant difference between oils, of which the highest percentage was recorded in *S*. *marianum* seed oil (16.98±10.96% and 13.09±4.46%, respectively).

**Table 1:** Yield oil contents of *S*. *marianum* and S. *eburneum seeds*.

Seeds	Oil contents (%)
***S*. *marianum***	27.76
***S*. *eburneum***	28.55

**Table 2:** Fatty acids profile of *S*. *marianum*, *S*. *eburneum*, and *S*. *marianum* commercial seed oils (%).

Fatty acids	*S*. *marianum*	*S*. *eburneum*	*S*. *marianum* commercial (Compagnie des sens)
**SFA**			
***Lauric acid (C12*:*0)***	0.738±0.728^a^	0.678±0.668^a^	0.01±0.0005^a^
***Myristic Acid (C14*:*0)***	0.091±0.005^a^	0.090±0.036^a^	0.10±0.005^a^
***Pentadecylic acid (C15*:*0)***	0.024±0.004^a^	0.023±0.002^a^	0.01±0.0005^b^
***Palmitic acid (C16*:*0)***	13.090±4.463^a^	12.712±1.110^a^	9.12±0.456^a^
***Margaric acid (C17*:*0)***	0.074±0.025^a^	0.068±0.008^a^	0.08±0.004^a^
***Stearic acid (C18*:*0)***	5.299±0.099^b^	5.350±2.287^b^	13.51±0.675^a^
***Arachidic acid (C20*:*0)***	2.273±0.897^b^	2.012±0.698^b^	3.77±0.188^a^
***Heneicosanoic acid (C21*:*0)***	0.017±0.007^a^	0.020±0.010^a^	0.01±0.0005^a^
***Behenic acid (C22*:*0)***	1.317±0.978^b^	0.768±0.180^b^	2.69±0.134^a^
***Lignoceric acid(C24*:*0)***	0.277±0.173^b^	0.222±0.101^b^	0.73±0.036 ^a^
**∑SFA**	23.199±3.017^ab^	21.942±5.080^b^	30.030±1.501^a^
**UFA**			
**MUFA**			
***(Z)-7-hexadecenoic acid (C16*:*1 n-9)***	0.020±0.001^b^	0.008±0.008^c^	0.07±0.003^a^
***Palmitoleic acid (C16*:*1 n-7)***	0.085±0.008^a^	0.078±0.015^a^	0.01±0.0005^b^
***Margaric acid (C17*:*1)***	0.015±0.005^a^	0.018±0.008^a^	0.02±0.001^a^
***Oleic acid (C18*:*1 n-9)***	16.979±10.957^a^	13.282±4.377^a^	24.79±1.239^a^
***Trans-vaccenic acid (C18*:*1 n-7)***	0.888±0.075^a^	0.809±0.088^ab^	0.72±0.036^b^
***Cis-11-eicosenoic acid (C20*:*1 n-9)***	0.495±0.291^b^	0.372±0.095^b^	0.95±0.0475^a^
***C24*:*1 n-9***	0.034±0.024^a^	0.017±0.007^a^	0.01±0.0005^a^
**∑MUFA**	18.517±11.154^a^	14.584±4.187^a^	26.570±1.328^a^
**PUFA**			
***Linoleic acid (C18*:*2 n-6)***	54.725±4.710^a^	54.532±9.462^a^	37.37±1.868^b^
***α-linolenic acid (C18*:*3n-3)***	0.091±0.081^a^	0.211±0.201^a^	0.32±0.016^a^
***γ-linolenic acid (C18*:*3 n-6)***	0.990±0.980^a^	0.255±0.245^a^	0.01±0.0005^a^
***Dihomo-gamma-linolenic acid (C20*:*2 n-6)***	0.026±0.016^a^	0.030±0.020^a^	0.01±0.0005^a^
**∑PUFA**	55.832±5.625^a^	55.028±9.398^a^	37.710±1.885^b^
**∑UFA**	74.349±5.529^a^	69.613±13.586^a^	64.280±3.214^a^

Data are expressed as mean ± standard deviation (n = 3); Significantly different values in the same row (*p* <0.05; Duncan test); Lower-case letters (a; b) indicate similarity or difference (same letter: similarity; different letter: difference) SFA: saturated fatty acids; UFA: unsaturated fatty acids; MUFA: monounsaturated fatty acids; PUFA: polyunsaturated fatty acids.

Phytosterols, components within the unsaponifiable lipid fraction of foods, are acknowledged for their potential health benefits. These compounds mainly include β-sitosterol, campesterol, and stigmasterol. These phytosterols are essential constituents of plant cell membranes, particularly in vegetable oils found in nuts, grains, and seeds. Consequently, it became imperative to assess both the quantity and composition of sterols in the examined *Silybum* seed oils (Table 3). Information about phytosterols data is illustrated in S2 Table. Our result identified 15 sterols in the three studied oils. Although no significant difference was observed in the total sterol contents between them (*p* = 0.111), *S*. *marianum* commercial seed oil exhibited the highest overall content (4770.2±143.1 mg/Kg of oil). Moreover, the major component characterizing the seed oils was sitosterol ranging from 1511.82 to 1861.89 mg/Kg of oil in *S*. *marianum* and *S*. *eburneum*, respectively. On the whole, the main components identified were campesterol, Δ^7^ campesterol, Δ^7^ stigmasterol, sitosterol, stigmasterol, and β-amyrin displaying a highly significant difference between oils. The highest values for Δ^7^ campesterol, Δ^7^ stigmasterol, stigmasterol, and β-amyrin (140.93±4.23, 1333.32±40, 345.97±10.38, and 215.66±6.47, respectively) were found in *S*. *marianum* seed oil. Conversely, campesterol and sitosterol were found in higher quantities in *S*. *eburneum* seed oil (556.09±16.68 and 1861.89±55.86 mg/Kg of oil, respectively). On the other hand, a high level of cholesterol was also detected, while the smallest amount was recorded in *S*. *marianum* seed oil (323.11±9.69 mg/Kg of oil), followed by *S*. *marianum* commercial seed oil (560.54±16.82 mg/Kg of oil) and finally *S*. *eburneum* seed oil (556.09±16.68 and 646.17±19.39 mg/Kg of oil, respectively) with highly significant difference (*p* = 0.000). Other sterols i.e., Δ^5^ avenasterol, Δ^7^ avenasterol, and 24-methylene cycloartenol were detected with high levels in *S*. *eburneum* (178.07±5.34, 141.90±4.26, and 130.77±3.92 mg/Kg of oil, respectively). Contrarily, *S*. *marianum* commercial seed oil showed the highest content of citrostadienol (67.17±2.02 mg/Kg of oil) compared to wild oils.

**Table 3:** Phytosterol profile of *S*. *marianum*, *S*. *eburneum*, and *S*. *marianum* commercial seed oils (mg/Kg of oil).

Phytosterols	*S*. *marianum*	*S*. *eburneum*	*S*. *marianum* commercial *(Compagnie des sens)*
***Unknown 1***	ND	35.84±1.08 ^a^	18.38±0.55 ^b^
***Campesterol***	195.69±5.87 ^c^	556.09±16.68 ^a^	266.30±7.99 ^b^
***Stigmasterol***	345.97±10.38 ^a^	269.95±8.10 ^c^	301.94±9.06 ^b^
***Unknown 2***	14.49±0.43 ^b^	88.23±2.65 ^a^	91.57±2.75 ^a^
***Δ*^*7*^ *Campesterol***	140.93±4.23 ^a^	93.01±2.79 ^b^	135.71±4.07 ^a^
***Sitosterol***	1511.82±45.35 ^b^	1861.89±55.86 ^a^	1598.61±47.96 ^b^
***Δ*^*5*^ *Avenasterol***	64.85±1.95 ^b^	178.07±5.34 ^a^	63.11±1.89 ^b^
***β Amyrin***	215.66±6.47 ^a^	116.46±3.94 ^c^	151.93±4.56 ^b^
***Unknown 3***	63.06±1.89 ^a^	34.34±1.03 ^c^	47.83±1.43 ^b^
***Δ*^*7*^ *Stigmasterol***	1333.32±40.00 ^a^	365.82±10.97 ^c^	1167.76±35.03 ^b^
***Δ*^*7*^ *Avenasterol***	83.19±2.50 ^b^	141.90±4.26 ^a^	27.99±0.84 ^c^
***24-methylene cycloartenol***	85.77±2.57 ^c^	130.77±3.92 ^a^	94.14±2.82 ^b^
***Epoxysitosterol***	94.53±2.84 ^c^	166.41±4.99 ^b^	177.24±5.32 ^a^
***Citrostadienol***	27.64±0.83 ^c^	43.77±1.31 ^b^	67.17±2.02 ^a^
***Cholesterol***	323.11±9.69 ^c^	646.17±19.39 ^a^	560.54±16.82 ^b^
**Total content**	4500.00±135.00 ^a^	4728.72±141.86 ^a^	4770.20±143.11 ^a^

Data are expressed as mean ± standard deviation (n = 3); Significantly different values in the same row (*p* <0.05; Duncan test); Lower-case letters (a; b; c) indicate similarity or difference (same letter: similarity; different letter: difference)

**Fig 1:**
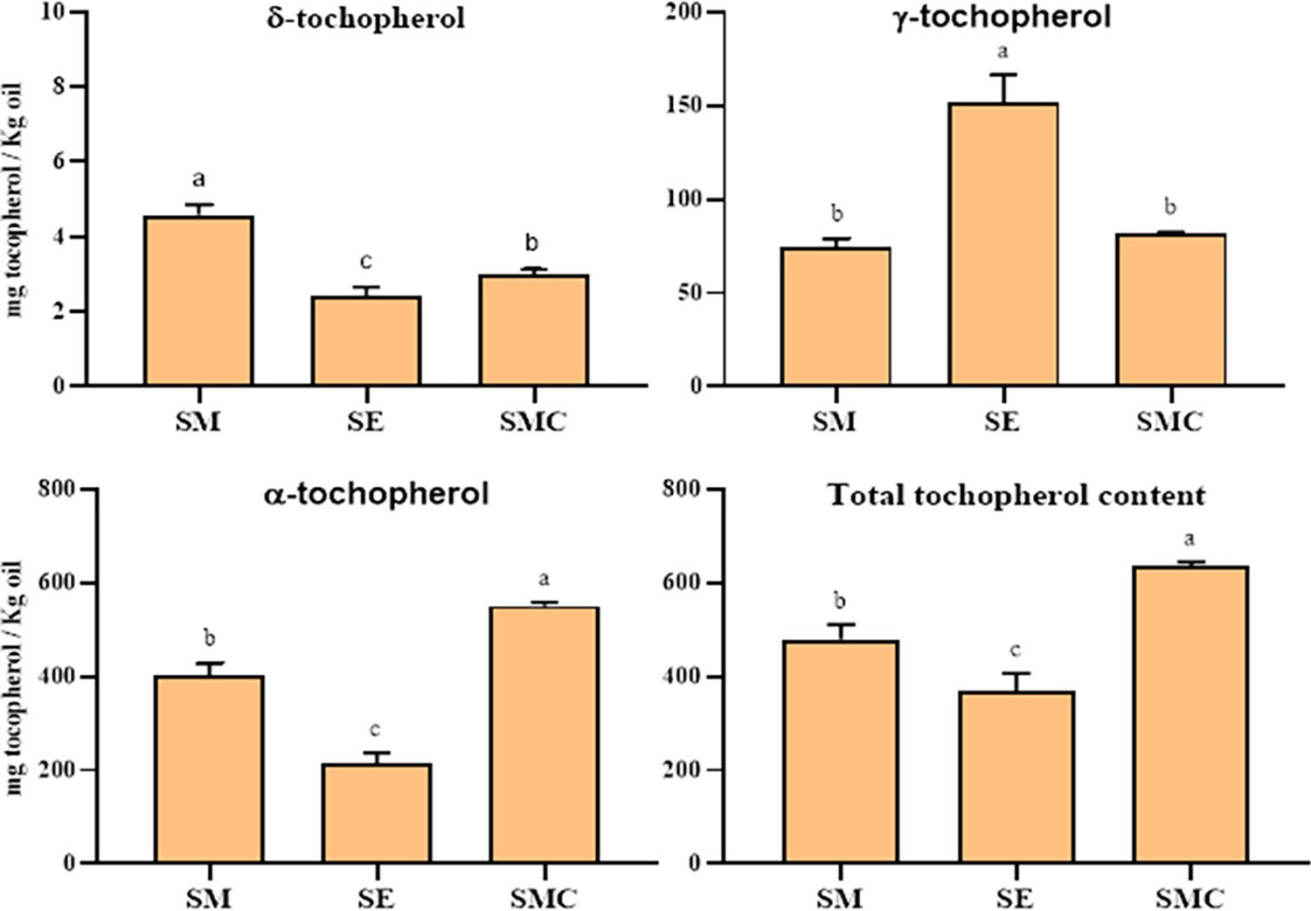
Tocopherols content of *S*. *marianum* (SM), *S*. *eburneum* (SE), and *S*. *marianum* commercial (SMC) seed oils (mg tocopherol/Kg of oil). Data are expressed as mean ± standard deviation (n = 3); Significantly different values in the same content (*p* <0.05; Duncan test for δ-tocopherol and Kruskal-Wallis test for γ, α, and total tocopherol content); Lower-case letters (a; b; c) indicate similarity or difference (same letter: similarity; different letter: difference).

The tocopherol content, including the δ-tocopherol, γ-tocopherol, α-tocopherol, and the total tocopherol content of SMSO, SESO, and SMCSO are presented in Fig 1 and the details of the data are exposed in S3 Table. The results showed that total tocopherol content varied significantly from 369.42±37.55 to 635.57±8.46 mg/Kg of oil for *S*. *eburneum* and *S*. *marianum* commercial seed oils, respectively. The α-tocopherol was the predominant form of tocopherols found in these oils, with the highest content observed in *S*. *marianum* commercial seed oil (551.001±7.23 mg/Kg of oil) followed by *S*. *marianum* and *S*. *eburneum* (400.833±26.59 and 215.55±21.99 mg/Kg of oil, respectively). In contrast, δ-tocopherol has the lowest tocopherol values in all oils (2.42±0.23, 3.002±0.13, and 4.58±0.28 mg/Kg of oil) for SESO, SMCSO, and SMSO, respectively.

### 3.2. Phenolic profiles and antioxidant activities of *S*. *marianum*, *S*. *eburneum*, and *S*. *marianum* commercial seed oils

The phenolic profile represents an important nutritional characterization for seed oil. Also, it gives a reflection about its antioxidant properties. The results, represented in Table 4, showed a significant difference in total flavonoids (*p* = 0.029) of the hydroalcoholic fraction of *S*. *marianum*, *S*. *eburneum*, and *S*. *marianum* commercial seed oil. The details of the data are filed in S4 Table. In contrast, there was no significant difference observed in total phenols and carotenoids content (*p* = 0.235 and *p* = 0.427, respectively). In particular, *S*. *marianum* seed oil has the highest levels of total flavonoids (20.87±1.74 mg QRE/100 g of oil) and carotenoids content (0.252±0.15 mg/kg of oil). However, the highest total phenols content was in *S*. *marianum* commercial seed oil (6.17±1.21 mg GAE/100 g of oil).

**Table 4:** Phenolic profile of *S*. *marianum*, *S*. *eburneum*, and *S*. *marianum* commercial seed oils.

	*S*. *marianum*	*S*. *eburneum*	*S*. *marianum* commercial (Compagnie des sens)
**Colorimetric quantification**			
***Total phenols content (mg GAE/100 g of oil)***	4.973±0.956^a^	4.792±0.572^a^	6.171±1.214^a^
***Total flavonoids content (mg QRE/100 g of oil)***	20.873±1.739^a^	16.226±1.664^b^	19.552±1.333^a^
***Total carotenoids content (mg/kg)***	0.252±0.152^a^	0.191±0.011^a^	0.226±0.033^a^
**HPLC quantification**			
**Phenolic acids** (mg equivalent Quercetin / 100 g of oil)			
***Vanillin***	0.13	0.28	0.06
***Coumarin***	0.43	ND	ND
***Silibinine***	ND	ND	0.36
**Peak at 48.1 min**	3.27	ND	ND
**Peak at 48.9 min**	5.48	ND	ND
**Sum peak at à 280 nm**	11.81	1.94	2.54

Data are expressed as mean ± standard deviation (n = 3) for colorimetric quantification. One measurement is realized for phenolic acids quantification by HPLC; significantly different values in the same row (*p* <0.05; Duncan test for total phenols and flavonoids content and Kruskal-Wallis test for total carotenoids content); Lower-case letters (a; b) indicate similarity or difference (same letter: similarity; different letter: difference). ND: not detected

In addition, the identification of phenolic acids by HPLC was also investigated and illustrated in Table 4. Only the vanillic acid was identified in all samples, which ranged between 0.06 and 0.13 mg QRE/100 g of oil in SMCSO and SMSO, respectively. Coumarin acid was identified only in *S*. *marianum* seed oil (0.43 mg QRE/100 g of oil), whereas, silibinine was only detected in *S*. *marianum* commercial seed oil (0.36 mg QRE/100 g of oil).

The richness of oils in total phenols and flavonoids confirms the important antioxidant activities found (Table 5). The ANOVA showed a significant difference between the three oils only for KRL and DPPH tests (*p* = 0.008 and *p* = 0.000, respectively). On the other hand, no significant difference was observed for the total antioxidant capacity (*p* = 0.140) and FRAP test (*p* = 0.123). Additionally, the highest total antioxidant activity, the DPPH radical scavenging, and the KRL test have been recorded for *S*. *marianum* seed oil (22.89±3.89 mg GAE/ 100 g of oil; 6.59±0.45 mg TRE / 100 g of oil; 237.79±23.93 mg TRE / g of oil and 95.36±9.47 mg GAE/ g of oil, respectively). On the other side, *S*. *eburneum* seed oil is more effective at the ferric reducing activity (0.81±0.55 mg TRE / 100 g of oil). For more details, data are exposed in S5 Table.

**Table 5:** Antioxidant activities of *S*. *marianum*, S. *eburneum*, and *S*. *marianum* commercial seed oils.

	Total antioxidant activity (mg GAE/ 100 g of oil)	DPPH (mg TRE / 100 g of oil)	FRAP (mg TRE / 100 g of oil)	KRL test	
				mg TRE / g of oil	mg GAE/ g of oil
***S*. *marianum***	22.890±3.89^a^	6.595±0.453^a^	0.589±0.078^a^	237.787±23.926^a^	95.363±9.469^a^
***S*. *eburneum***	17.029±4.257^a^	6.309±0.559^a^	0.807±0,549^a^	137.203±29.937^b^	55.003±11.807^b^
***S*. *marianum commercial (Compagnie des sens)***	17.537±0.885^a^	2.009±0.789^b^	0.173±0.018^a^	143.473±29.569^b^	62.460±9.053^b^

Data are expressed as mean ± standard deviation (n = 3); significantly different values in the same column (*p* <0.05; Duncan test); Lower-case letters (a; b) indicate similarity or difference (same letter: similarity; different letter: difference).

### 3.3. Cytoprotective effect of *S*. *marianum*, *S*. *eburneum*, and *S*. *marianum* commercial seed oils

#### 3.3.1. Toxicity effect of *S*. *marianum*, *S*. *eburneum*, and *S*. *marianum* commercial seed oils on THP-1 cells

**Fig 2:**
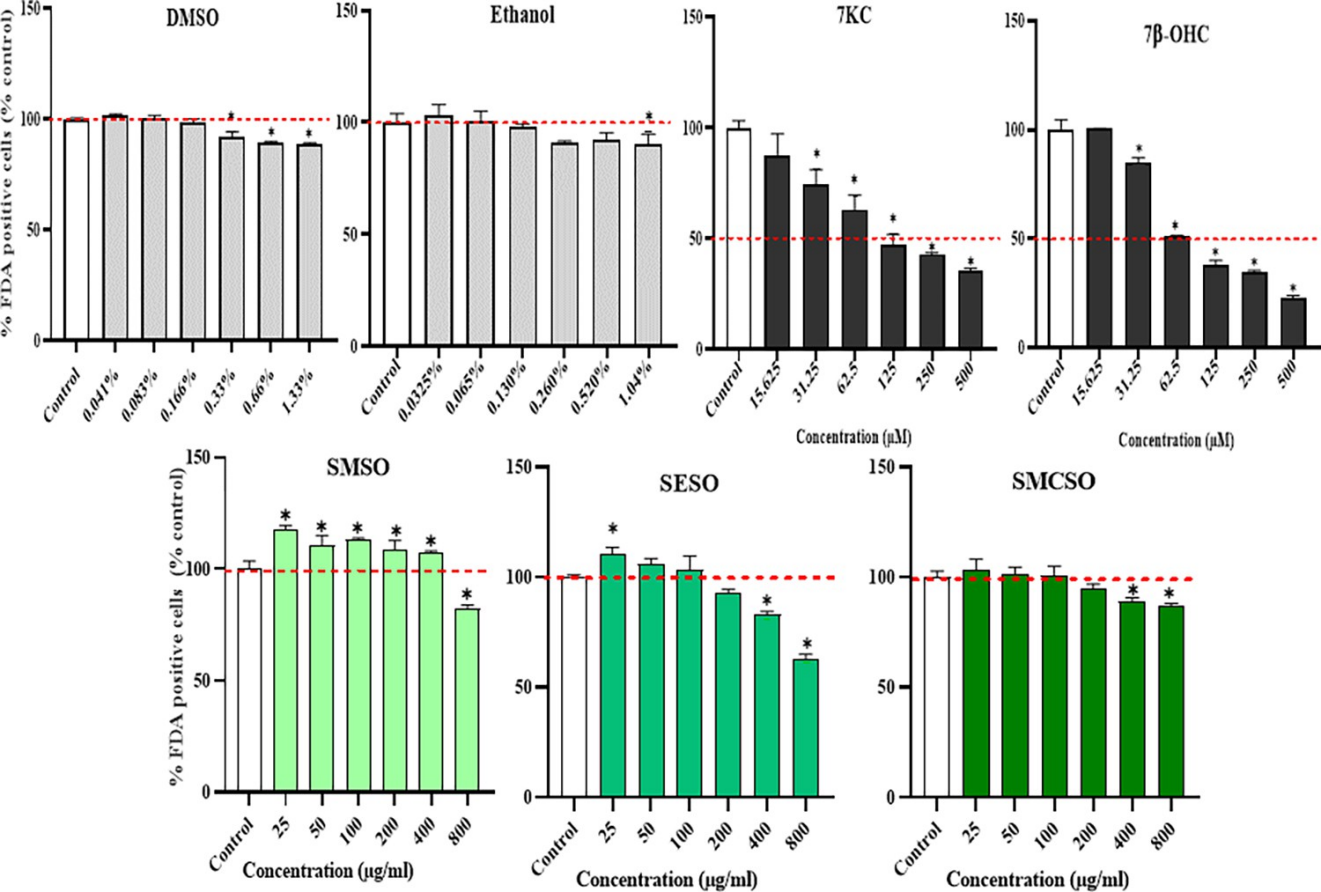
Evaluation of the cytotoxic effects of *Silybum* seed oils (SMSO, SESO, and SMCSO) and oxysterols (7KC, 7β-OHC) on THP-1 cells with the FDA test after 24 hours of treatment. The histograms represent the percentage of FDA-positive cells considered alive compared to the control. The results are expressed as mean ±SD of an experiment performed in triplicate. (*) indicate significative difference port to control (*p* <0.05; Bonferroni’s test). 7KC: 7-cetocholesterol; 7β-OHC: 7β-hydroxycholesterol; SMSO: *S*. *marianum* seed oil; SESO: *S*. *eburneum* seed oil, and SMCSO: *S*. *marianum* commercial seed oil.

Firstly, experiments were carried out on THP-1 cells to assess whether vehicle (DMSO and ethanol), oxysterols (7KC and 7β-OHC), and the oils (SMSO, SESO, and SMCSO) alone affect cell death. The results, illustrated in Fig 2, showed that there was no toxic effect of oils and vehicles compared to untreated THP-1 cells. Additionally, the percentage of viable cells compared to control decreased to 82.12%, 93.12%, and 94.72% for SMSO (800 μg/mL), SESO (200 μg/mL), and SMCSO (200 μg/mL), respectively. Furthermore, in the presence of 7KC and 7β-OHC at a concentration of 62.5 μM, the percentages of viable THP-1 cells reduced to 63.10% and 50.98%, respectively. On the other hand, a significant effect was observed in DMSO from 0.33% (92.28% of viable cells compared to the control) and only in 1.04% for ethanol (90.14% of viable cells compared to the control). Further experiments were conducted based on these results, using oils at a concentration of 100 μg/ml (0.166% of DMSO) and oxysterols at 62.5 μM (0.130% of ethanol), which showed no toxicity toward THP-1 cells. The data details can be found in S6 Table.

#### 3.3.2. Effects of *S*. *marianum*, *S*. *eburneum*, and *S*. *marianum* commercial seed oils on 7-ketocholesterol and 7β-hydroxycholesterol induced oxidative ROS overproduction in THP-1 cells

**Fig 3:**
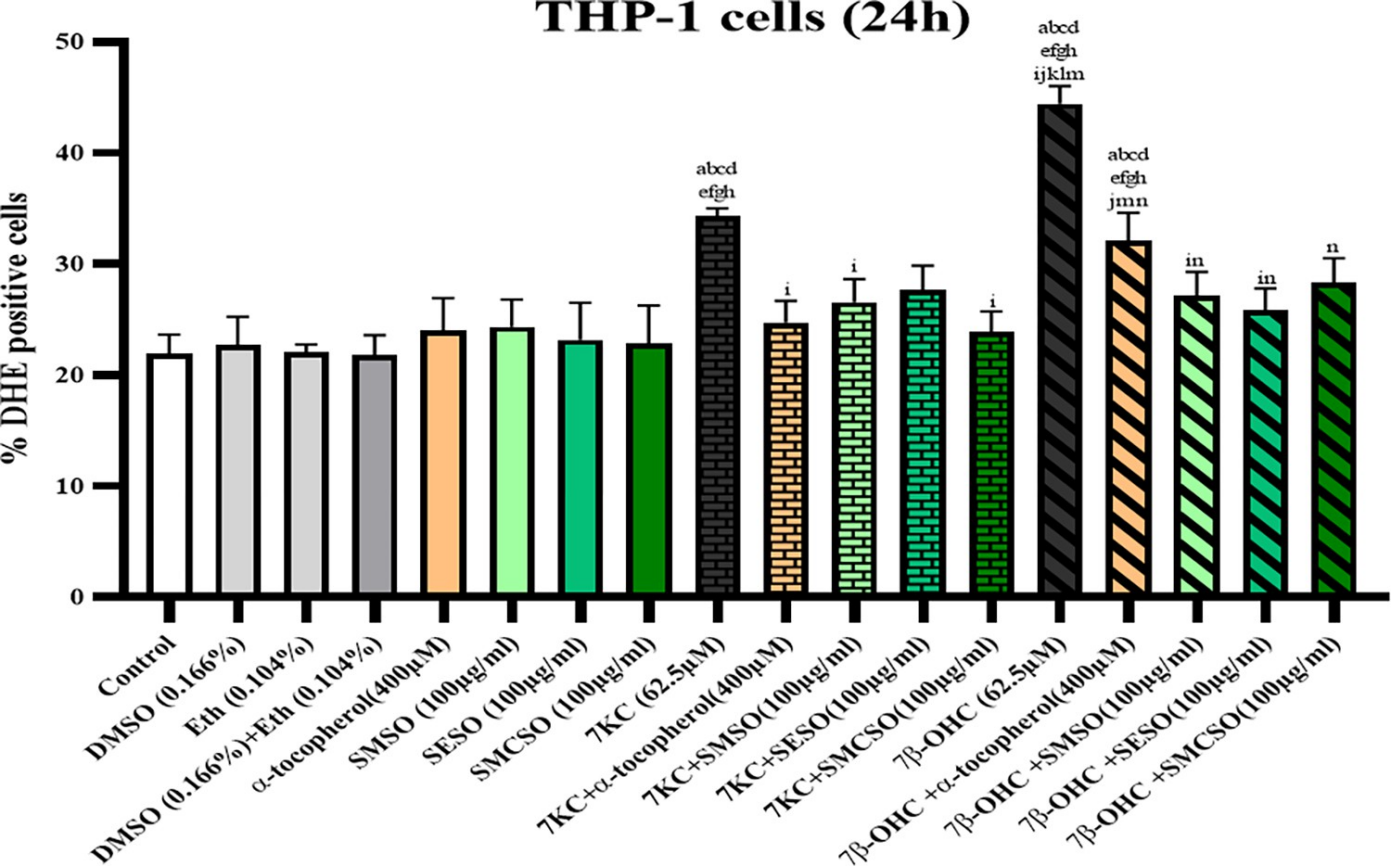
Effects of *S*. *marianum*, *S*. *eburneum*, and *S*. *marianum* commercial seed oils on oxysterols (7-cetocholesterol and 7β-hydroxycholesterol) induced ROS overproduction in THP-1 cells. THP-1 cells were incubated for 24 h with or without 7KC et 7β-OH (62.5μM) in the presence or absence of *Silybum* seed oils (SMSO, SESO, and SMCSO) (100 μg/mL) or α-tocopherol (400 μM). The histogram represented the percentage of DHE-positive cells. The results are expressed as mean ±SD of three independent experiments. Different letters indicate significate difference between the groups (*p* <0.05, Tukey test, multiple comparative analysis). a: comparison to control; b: comparison to DMSO (0.166%); c: comparison to Eth (0.104%); d: comparison to “DMSO (0.166%) +Eth (0.104%)”; e: comparison to α-tocopherol (400 μM); f: comparison to SMSO (100 μg/mL); g: comparison to SESO (100μg/mL); h: comparison to SMSO (100 μg/mL); i: comparison to 7KC (62.5 μM); j: comparison to “7KC + α-tocopherol (400 μM)”; k: comparison to “7KC + SMSO (100 μg/mL)”; l: comparison to “7KC + SESO (100 μg/mL)”; m: comparison to “7KC + SMCSO (100 μg/mL)”; n: comparison to 7β-OH (62.5 μM); o: comparison to “7β-OH + α-tocopherol (400 μM)”; p: comparison to “7β-OH + SMSO (100 μg/mL)”; q: comparison to “7β-OH + SESO (100 μg/mL)”; r: comparison to “7β-OH + SMCSO (100 μg/mL)”.

To study the impact of SMSO, SESO, and SMCSO (100 μg/mL) on ROS overproduction in THP-1 cells treated with 7KC and 7β-OH (62.5 μM, 24h), we measured ROS production by flow cytometry after staining with dihydroethidium (DHE). Fig 3 shows no significant difference between the control group (untreated cells), the vehicle group (cells treated with DMSO and ethanol) and the groups treated with α-tocopherol (a positive control used as an antioxidant molecule) and oils (SMSO, SESO, and SMCSO). However, there was a highly significant increase in DHE-positive cells in cells treated with 7KC and 7β-OH (62.5 μM) compared to the control, vehicles, α-tocopherol, and oils (*p*<0.0001). These results indicate that 7β-OH induces more oxidative stress than does 7KC inTHP-1 cells. The percentage of DHE-positive cells for 7β-OH was 44.45±1.55%, while for 7KC it was 34.3±0.72%, indicating a highly significant difference (*p* = 0.0003). The overproduction of ROS decreased when 7KC and 7β-OH were combined with α-tocopherol and the three oils. The study found that SMCSO was the most effective in reducing the oxidative damage of 7KC on THP-1, with a significant reduction of 30.38% compared to 7KC. α-tocopherol and SMSO also showed a significant reduction of 27.87% and 22.51%, respectively. However, SESO only resulted in a non-significant reduction of 19.27% (*p* = 0.0652). On the other hand, a highly significant difference was shown between cells treated with 7β-OH alone and those associated with α-tocopherol, SMSO, SESO and SMCSO. SESO showed the highest reduction rate (41.96% redaction compared to 7β-OH), followed by SMSO and SMCSO (38.81% and 36.33%, respectively). α-tocopherol also showed a capacity to reduce ROS production, with a redaction rate of 27.78% compared to cells treated with 7β-OH only. Refer to S7 Table for data details.

### 3.4. Principal component analysis (PCA) and heatmap

**Fig 4:**
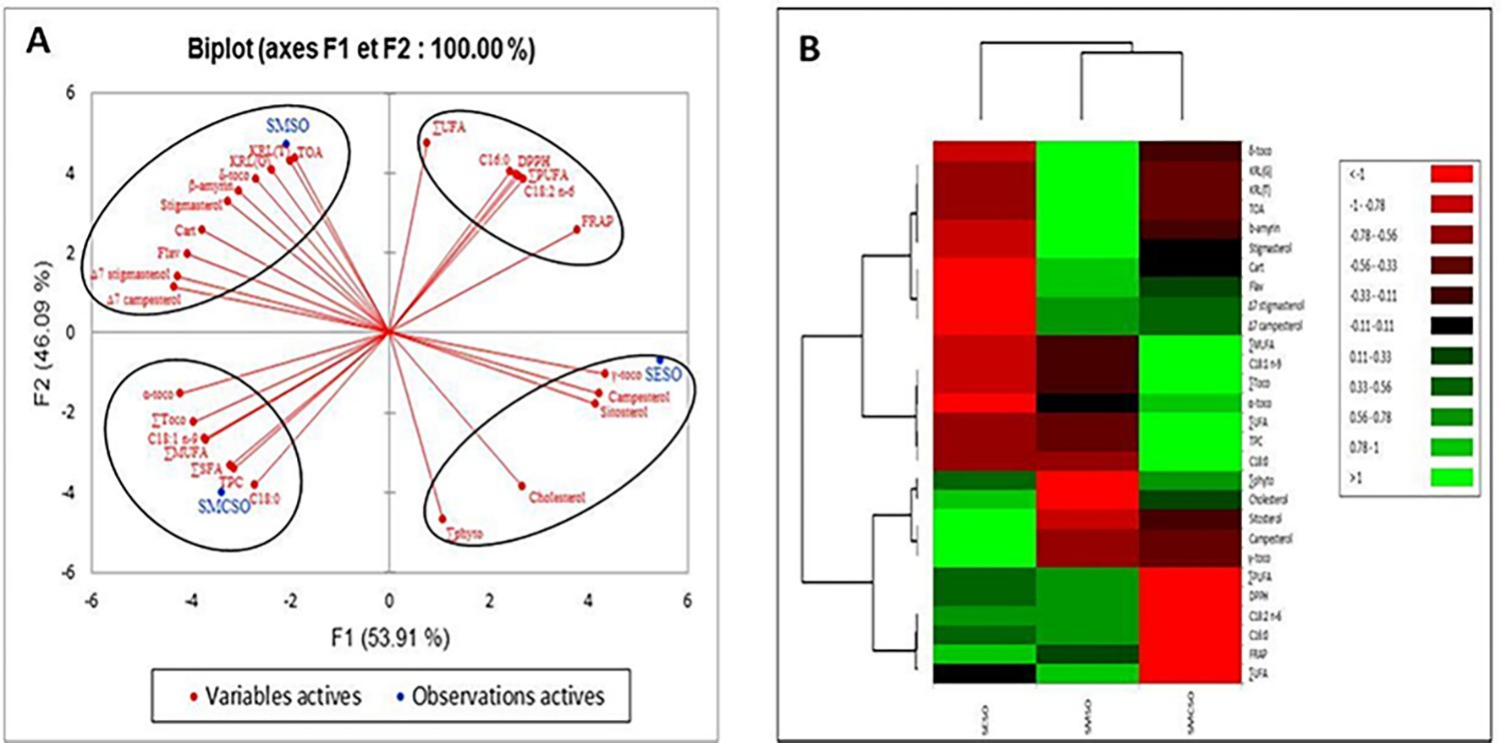
Principal component analysis (PCA) (A) and heatmap (B) of lipid profiles (fatty acids, phytosterols, and tocopherols), phenolic profiles (total phenol, flavonoid, and carotenoid contents), and antioxidant activities (DPPH, FRAP, TOA, and KRL) of the three oils. TPC: total phenolic content; Flav: total flavonoid content; Cart: total carotenoid content; TOA: total antioxidant activity; KRL (G): garlic acid equivalence; KRL (T): Trolox equivalent; δ-toco: δ-tocopherol; γ-toco: γ-tocopherol; α-toco: α-tocopherol; ∑Toco: total tocopherol content; C16:0: palmitic acid; C18:0: stearic acid; ∑UFA: unsaturated fatty acids; C18:1n-9: oleic acid; ∑MUFA: mono-unsaturated fatty acids; C18:2 n-6: linoleic acid; ∑PUFA: polyunsaturated fatty acid; ∑UFA: unsaturated fatty acids; ∑phyto: total phytosterol content; SMSO: *S*. *marianum* seed oil; SESO: *S*. *eburneum* seed oil; SMCSO: *S marianum* commercial seed oil.

Fig 4A presents the biplot of the principal component analysis (PCA), which was carried out to compare the lipid profiles (fatty acids, phytosterols and, tocopherols), phenolic profiles (total phenol, flavonoid and, carotenoid contents) and, antioxidant activities (DPPH, FRAP, KRL test and, total antioxidant activity) of the three oils: *S*. *marianum* seed oil, *S*. *eburneum* seed oil and, *S*. *marianum* commercial seed oil. The analysis included two components: PC1 and PC2. PC1 accounted for 53.91% of the variance and was dominated by total flavonoid and carotenoid contents, FRAP, γ-tocopherol, α-tocopherol, total tocopherol content, total saturated fatty acid, C18:1 n-9, total monounsaturated fatty acid, campesterol, stigmasterol, ∆^7^ campesterol, sitosterol, and ∆^7^ stigmasterol. On the other hand, the second component (PC2) represented 46.09% of the variability and was dominated by δ-tocopherol, total phenol content, total antioxidant activity, DPPH, KRL test, C16:0, C18:0, C18:2 n-6, total polyunsaturated fatty acids, unsaturated fatty acids, β-amyrin, cholesterol, and total phytosterol compounds. The hierarchical cluster (Fig 4B), showed four distinct groups. The first group demonstrated a strong correlation between SMCSO and C18:0, C18:1 n-9, total phenol content, saturated fatty acid, unsaturated fatty acid, total tocopherol content, and α-tocopherol. Meanwhile, SMSO was found to be correlated with ∆^7^ campesterol, ∆^7^ stigmasterol, stigmasterol, β-amyrin, δ-tocopherol, total antioxidant activity, KRL test, and total flavonoid and carotenoid contents in the second group. The high antioxidant activity of SMSO may be attributed to its richness in phytosterols, tocopherols, and phenolic compounds which are known for their high antioxidant activity. The third group included unsaturated fatty acids, polyunsaturated fatty acids, C16:0, C18:2 n-6, DPPH, and FRAP. This group was equally distant from the SMSO and SESO. The correlations between SESO and γ-tocopherol, sitosterol, campesterol, cholesterol, and total phytosterol were presented by the fourth group.

The variables with the highest correlation to each oil indicate that this oil contains a greater quantity of these variables compared to the other oils. These findings are further supported by the heatmap shown in Fig 4B where the oil with the highest content of variable is indicated by the green color. For additional information on the data, refer to S8 Table.

## 4. Discussion

Wild plants are considered a significant source of bioactive compounds with medicinal and therapeutic properties. As part of the development of these plants, we focused on the *Silybum* species. *S*. *marianum* is a Mediterranean spontaneous edible plant and the only known representative of the genus *Silybum*. Nevertheless, some authors mention a second species, *S*. *eburneum*, which is considered a marginalized species. The biological and biochemical characterization of *S*. *eburneum* seed oil have not been studied to date, compared with *S*. *marianum*. Therefore, the study aimed to evaluate the fatty acid, phytosterol, and tocopherol profiles of *S*. *marianum*, *S*. *eburneum*, and *S*. *marianum* commercial seed oils to compare the biological and biochemical characterization of these oils. The total phenolic, flavonoid, and carotenoid contents, the polyphenolic profiles, and the antioxidant properties of these oils were also studied as well as the effect of these oils on ROS overproduction of human monocytic THP-1 cells. The different data obtained support that this oil has much nutritional interest which is in the range of those previously reported for olive oil and argan oil [22].

One of the most important characteristics of seeds is their percentage of oil content (%). In this study, the content varied between 27% and 30% for *S*. *marianum* and *S*. *eburneum* seeds, respectively, in which we can consider the milk thistle seeds as oil seeds. These percentages are higher than those registered in Polish *S*. *marianum* seed oil using the supercritical CO_2_ method (26% of oil) [20] and those of two Greece varieties showing oil contents ranging between 22.1% and 25.1% [19]. Similarly, the content of *S*. *marianum* seed oil collected from Sousse-Tunisia was around 30.5% [18] and 31.83% in seeds collected from North-Tunisia [9]. Likewise, the oil content of 30 Greek populations of *S*. *marianum* varied between 24.65% and 31.10% [17]. Consequently, oil content depends on germplasm and environmental conditions [54].

Fatty acids are biochemical compounds that play important roles in human metabolism and individual health [55]. The composition of fatty acids in vegetable oils reflects their nutritional quality. In this study, the oils were rich in unsaturated fatty acids (UFA), which varied from 64.28% to 74.35%. These findings are consistent with the levels of UFA found in milk thistle oil [21]. On the other hand, a cold press extraction of Tunisian *S*. *marianum* seeds showed that UFA content varied from 79.12% (for seeds from Bizerte) to 83.3% (for seeds from Zagouan) [8].

The polyunsaturated fatty acids (PUFA) content (varied from 37.71% to 55.83%) is higher than monounsaturated fatty acids (MUFA) content (between 14.58% and 26.57%). These values are close to those found by Harrabi *et al*. [18], who mentioned the richness of Tunisian *S*. *marianum* seed oil with PUFA (60.37%). This high level of unsaturated fatty acids in milk thistle seed oils increases their susceptibility to oxidation. The major UFA identified in this study were the linoleic acid (C18:2 n-6) and the oleic acid (C18:1 n-9). Linoleic acid (omega-6) is an essential fatty acid that has a beneficial effect on human health. Incorporating it into our diet can contribute to lowering the risk of cardiovascular disease [23, 56, 57]. Similarly, oleic acid is a major fatty acid found in olive oil. It inhibits the biosynthesis of fatty acids and helps the protection against cardiovascular disease [24]. In this study, the linoleic acid values ranged from 37.37% to 54.72% while the oleic acid content varied between 13.28% and 24.79%. Additionally, palmitic acid (C16:0) levels ranged from 9.12% to 13.09% followed by stearic acid (C18:0; 5.30–13.51%). These contents are higher than those found in Iranian *S*. *marianum* seed oil [29]. In addition, Harrabi *et al* showed that the Tunisian *S*. *marianum* seed oil was rich in linoleic acid (59.98%) followed by oleic acid (21.26%) and palmitic acid (12.74%) [18]. In line with these findings, Zarrouk *et al*. noted that the predominant fatty acids in the oil of three accessions of milk thistle gathered from three Tunisian regions (Zaghouane, Bizerte, and Sousse) were linoleic (C18:2 n-6; 48.70–58.47%), oleic (C18:1 n-9; 15.78–21.39%), palmitic (C16:0; 6.25–13.06%), and stearic (C18:0; 3.35–5.21%) acids [22]. Moreover, many other authors have indicated that *S*. *marianum* seed oil is rich in linoleic acid, oleic acid, and palmitic acid [8, 26, 58].

In addition to the fatty acid profile, we studied another significant profile that contributes to the characterization of vegetable oils, which is the sterols or phytosterols profile. Phytosterols are commonly found in most vegetable oils and are structurally similar to cholesterol, differing only in the side chain attached to the steroid ring. They are known for their ability to lower plasma low-density lipoprotein cholesterol (LDL-C) and exhibit anti-inflammatory properties [24].

In this study, the total phytosterol content ranged from 4500 to 4770.2 mg/Kg of oil. This content exceeds that identified by Marszałkiewicz *et al*, which ranged between 2910 and 3040 mg/Kg of oil [26]. Nevertheless, it is similar to *S*. *marianum* seed oil from a different region of Tunisia, as described by Meddeb *et al*. and Zarrouk *et al*. [8, 22].

The major constituents detected in this study were sitosterol (1511.82–1861.89 mg/Kg), followed by Δ7 stigmasterol (365.82–1333.32 mg/Kg), both accounting for 33.51% - 39.37% and 7.74% - 29.63% of the total phytosterol content, respectively. Other phytosterols were also found: campesterol (195.96 to 556.09 mg/Kg), stigmasterol (269.95 to 345.97 mg/Kg), Δ7 campesterol (93.01 to 140.93 mg/Kg), and β-amyrin (116.46 to 215.66 mg/Kg). Additionally, cholesterol was identified, with a percentage ranging between 7.18% and 13.66% of the total phytosterol content (323.11 to 646.17 mg/Kg). Moreover, other cholesterols were found in almost significant quantities, such as 24-methylene cycloartenol, epoxy sitosterol, Δ5 avenasterol, and Δ7 avenasterol. However, citrostadienol showed the least contribution.

In general, plants contain three representative phytosterols: campesterol, sitosterol, and stigmasterol [59]. The detected phytosterols were consistent with the findings of Zarrouk *et al*, who reported Tunisian SMSO phytosterol content ranging from 4.53% to 11.47% of cholesterol, with the major phytosterols detected being β-sitosterol, schotenol, and stigmasterol [22]. However, it should be noted that the phytosterols present in Iranian *S*. *marianum* seed oil are predominantly 4-desmethylsterol, 4,4’-dimethylsterols, and 4-monomethylsterols, as identified by Khallouki *et al*. [29]. Conversely, in *S*. *marianum* seed oil from Poland and Russia, six major phytosterols have been detected, namely campesterol, stigmasterol, β-sitosterol, avenasterol, Δ7 stigmasterol, Δ7 avenasterol, and cholesterol, as reported by Marszałkiewicz *et al*. [26]. Also El-Mallah *et al*. detected campesterol, 5-stigmasterol, β-sitosterol, 7-stigmasterol, avenasterol, and spinasterol in milk thistle seed oil [25].

Campesterol is a dietary supplement that has been shown to offer numerous health benefits. It can reduce cholesterol levels, improve heart health, reduce inflammation, and improve blood sugar levels. According to Luz Fernandez and Vega-López, sitosterol is a beneficial dietary supplement for reducing plasma cholesterol levels [60]. However, caution should be exercised when administering sitosterol to individuals with a higher absorption rate of sitosterol. Several investigations have demonstrated that stigmasterol possesses significant anticancer properties against various tumor cell lines. It has also been found to exhibit pharmacological effects such as anti-osteoarthritis, anti-inflammatory, anti-diabetic, immunomodulatory, antiparasitic, antifungal, antibacterial, antioxidant, and neuroprotective properties [61].

The content of tocopherols, including δ-tocopherol, γ-tocopherol, and α-tocopherol, as well as the total tocopherol content, was also investigated in this study. Tocopherols are a form of vitamin E [24] and have many biological activities, particularly α-tocopherol, which acts as a non-enzymatic antioxidant that interrupts the chain of oxidation and peroxidation in aerobic organisms [62].

In the present study, we also report that the oils are rich in α-tocopherol, ranging from 215.55 to 551 mg/Kg of oil, followed by γ-tocopherol (74.54 to 151.44 mg/Kg) and δ-tocopherol (2.42 to 4.58 mg/Kg). These results are consistent with the findings of Marszałkiewicz *et al*. who found that the most dominant form of tocopherol in milk thistle seed oil from Poland and Russia is α-tocopherol, with a value ranging from 466.8 to 503.9 mg/Kg [26]. Similarly, Bahram and Sodeif showed that the levels of α-, β-, γ-, and δ-tocopherols in Iranian *S*. *marianum* seed oil ranged from 187 to 465, from 10 to 51, from 9 to 12, and from 18 to 80 μg/g oil, respectively [29]. However, the tocopherol levels of *S*. *marianum* seed oil from various regions of Tunisia, as described by Meddeb *et al*. and Zarrouk *et al*., were found to be lower than our results [8, 22].

In addition to the lipid profiles of the three *S*. *marianum* seed oils, including fatty acids, phytosterols, and tocopherols, we investigated their phenolic profile, including total phenols, flavonoids, carotenoids contents, and phenolic acids, as well as their antioxidant activities (TOA, DPPH, FRAP, and KRL). Phenolic compounds constitute the largest family of secondary metabolites produced by plants, with flavonoids being the most abundant among them. Flavonoids are known for their various bioactive effects, including anti-inflammatory, anti-cancer, cardioprotective, anti-diabetic, anti-viral, and anti-aging properties [63]. Additionally, carotenoids (or vitamin A) play a crucial role in maintaining human health. Research suggests that they offer a range of benefits, including promoting eye health, enhancing cognitive function, improving cardiovascular health, and potentially reducing the risk of certain types of cancer [64].

The hydroalcoholic fractions of *S*. *marianum*, *S*. *eburneum*, and *S*. *marianum* commercial seed oils contained varying amounts of total phenols, flavonoids, and carotenoids, ranging from 4.79 to 6.17 mg GAE/100 g, 16.23 to 20.87 mg QRE/100 g, and 0.19 to 0.25 mg/Kg, respectively. Similarly, Meddeb *et al*. found that the total polyphenol content ranged from 3.59 to 8.12 mg GAE/g of oil [7]. On the other hand, the cold press of Jordanian *S*. *marianum* seed oil showed a value of ≈ 1.16 mg GAE/g oil [2]. Furthermore, the cold-pressed *S*. *marianum* seeds exhibited a total phenol content of 3.01 mg GAE/g oil and a carotenoid content of 2.3 μM/Kg oil [65].

For the identification of phenolic acids by HPLC, only vanillic acid was identified in all samples, while p-coumaric acid and silibinine were only detected in SMSO and SMCSO, respectively. These results are comparable to those reported by Meddeb *et al*., who found only p-coumaric acid in the seed oil of *S*. *marianum* from three Tunisian regions; in contrast, vanillic acid was detected in *S*. *marianum* seed oil from Sousse and Zaghouane [8]. On the other hand, Zarrouk *et al*. detected homovanillic acid, vanillic acid, p-coumaric acid, quercetin-3β-glucoside acid, quercetin acid, and apigenin acid in the seed oil of *S*. *marianum* from Zaghoune and Bizerte, and only vanillic acid was detected in *S*. *marianum* seed oil from Sousse [22]. RP-HPLC analysis identified silybin A, silybin B, isosilybin A, isosilybin B, silychristin, and silydianin as the phenolic compounds present in Pakistani *S*. *marianum* seed oil [58]. Also, Hammouda *et al*. identified only quercetin and apigenin acids in *S*. *marianum* seed oil [6]. This variation in detected phenolic acids may be attributed to the HPLC library standard used, the origin of seeds, and the oil extraction methods.

The richness in total phenols and flavonoids confirms the important antioxidant activities found. In fact, four types of antioxidant tests were analyzed in this study: total antioxidant activity, DPPH, FRAP, and KRL tests [47]. We found that the total antioxidant activity of SMSO, SESO, and SMCSO ranged between 17.03 and 22.89 mg GAE/100 g of oil. The DPPH test showed a variation from 2.009 to 6.595 mg TRE/100 g of oil. However, the FRAP test recorded low activity, with values ranging from 0.173 to 0.807 mg TRE/g of oil. Furthermore, the KRL test showed significant activity with values ranging from 137.20 to 237.79 mg TRE/g oil and from 55.003 to 95.363 mg GAE/g oil.

The results obtained in this study are lower than those reported by Zarrouk *et al*. for the KRL activity of Tunisian *S*. *marianum* seed oils, which ranged from 2742 to 8228 mg Trolox/mL of oil and from 1042 to 3126 mg GAE/mL oil [22]. Similarly, Meddeb *et al*. reported that the DPPH, FRAP, and KRL values of SMSO ranged from 98.54 to 216.16 Trolox equivalent/mL of oil, from 122.14 to 211.06 Trolox equivalent/mL of oil, and from 144.25 to 180.48 Trolox equivalent, respectively [8]. However, in contrast to the above findings, Dabbour *et al*. reported that the IC50 value of Jordanian *S*. *marianum* seed oil in the DPPH test was 3.34 mg/mL [2]. Similarly, Hammouda *et al*. found that Tunisian *S*. *marianum* seed oil demonstrated significant antioxidant activity with EC50 values of 4.30 and 4.53 mg/mL of oil for DPPH and FRAP tests, respectively [6].

This variation in lipid and phenolic profiles and antioxidant activities of SMSO, SESO, and SMCSO could be explained by the method of extraction of oil and the polyphenol fraction, as well as the climatic conditions of the region where the seeds were collected.

However, the high levels of unsaturated fatty acids (USFA), phytosterols, α-tocopherols, carotenoids, flavonoids, and phenols in the seed oils in our study, along with their significant antioxidant activities, have piqued our interest in investigating the in vitro oxidative stress effect on THP-1 cells. We evaluated the toxicity effect of the seed oils on THP-1 cells with the FDA test (cell viability test). The data show that the *Silybum* seed oils did not have a toxic effect on THP-1 cells treated for 24 h with 100 μg/ml of oil. The *S*. *marianum* seed oil has a similar effect on other cells such as, murine oligodendrocytes cells (158N), murine microglial (BV2), murine myoblasts (C2C12), and human neuroblastoma cells (SH-SY5Y) [5, 6, 8, 22]. On the other hand, a toxic effect appeared when THP-1 was treated with 7KC and 7β-OH.

The monocyte cell line THP-1 was one of the line cells used as an immune model and also to study inflammation and ROS overproduction [66, 67]. ROS overproduction in cells can be influenced by oxysterols. The auto-oxidation of cholesterol can produce two major oxysterols which are 7-ketocholesterol and 7β-hydroxycholesterol [41]. In addition, many bioactive compounds such as tocopherols, fatty acids, phytosterols, and polyphenols or vegetable oils in general can reduce the damaging effect of oxysterols [5, 6, 44, 68]. In this context, we investigate the overproduction of ROS in THP-1 incubated for 24h with or without 7KC et 7β-OH (62.5μM) in the presence or absence of *Silybum* seed oils (SMSO, SESO, SMCSO) (100μg/mL) or α-tocopherol (400 μM). Treatments with 7KC and 7β-OH resulted in a noteworthy rise in the proportion of DHE-positive cells in comparison to control (untreated cells), vehicles (DMSO and ethanol), oils (SMSO, SESO, and SMCSO), and α-tocopherol treated cells. In light of these results, Brahmi *et al*. demonstrated that 7KC contributes to the development of oxidative stress in human macrophage and monocyte cells such as U937 and THP-1 [68]. Furthermore, our results demonstrated that 7β-OH is more toxic than 7KC in terms of the overproduction of ROS in THP-1 cells. Several studies demonstrate that 7β-OH was the highest cytotoxic oxysterol in different cell lines [40, 41, 44, 69]. In this study, the increase in ROS generation was mitigated by the association of 7KC and 7β-OH with oils (SMSO, SESO, and SMCSO) and α-tocopherol (positive control). Similarly, Hammouda *et al* showed that Tunisian *S*. *marianum* seed oil was able to reduce lipid and protein oxidation induced by 7β-OH on SH-SY5Y cells [6]. However, treatment of murine C2C12 myoblasts cells with 7β-OH in combination with *S*. *marianum* seed oil resulted in a reduction in DHE-positive cells compared to cells treated with 7β-OH alone [5]. Additionally, the overproduction of ROS was significantly reduced when Tunisian *S*. *marianum* seed oil was combined with 7KC and 24S-OHC, in 158N murine oligodendrocyte cells, compared to that in cells treated with 7KC and 24S-OHC alone [8].

## 4. Conclusion

This work is the first report which gives simultaneous information on *S*. *eburneum* seed oil comparative to *S*. *marianum* seed oil. The present study shows that *S*. *marianum*, *S*. *eburneum*, and *S*. *marianum* commercial seed oils contain high amounts of unsaturated fatty acids, phytosterols, and tocopherols which are major nutrients in the human diet. These oils also contain significant amounts of phenolic compounds. These oils have also important antioxidant activities, as determined by the FRAP, DPPH, and KRL tests. In human monocytic THP-1 cells, all the oils studied strongly reduce ROS overproduction induced by 7KC and 7β-OH which are strongly pro-oxidant oxysterols identified at increased levels in several major age-related diseases. On the THP-1 cells, *S*. *eburneum* seed oil demonstrated the greatest activity. Taken together, the data obtained support that *S*. *marianum* and *S*. *eburneum* seed oils are important sources of bioactive molecules with nutritional interests to prevent age-related diseases whose frequency is increasing in all countries due to the length of life expectancy whatever the countries considered.

## Supporting information

S1 Table Data of fatty acids profile of the three oils. (PDF)

S2 Table Data of phytosterol profile of the three oils. (PDF)

S3 Table Data of tocopherol content of the three oils. (PDF)

S4 Table Data of phenolic profile of the three oils. (PDF)

S5 Table Data of antioxidant activities of the three oils. (PDF)

S6 Table Data of FDA test. (PDF)

S7 Table Data of DHE test. (PDF)

S8 Table Data of principal component analysis (PCA) and heatmap tests. (PDF)

## Abbreviations

7β-OH: 7β-Hydrocholesterol
7KC: 7-ketocholesterol
DHE: Dihydroethidium
DMSO: Dimethyl sulfoxide
DPPH: 2,2-diphenyl 1-picrylhdrazyle
FDA: Fluorescein Diacetate Assay
FRAP: Ferric Reducing Antioxidant Power
GAE: Gallic acid equivalent
KRL: Kit Radicaux Libres
MUFA: Monounsaturated Fatty Acids
PUFA: Polyunsaturated Fatty Acids
QRE: Quercetin Equivalent
ROS: Reactive oxygen species
RPMI 1640: Roswell Park Memorial Institute 1640
SE: *Silybum eburneum*
SESO: *Silybum eburneum* seed oil
SFA: Saturated Fatty Acid
SM: *Silybum marianum*
SMC: *Silybum marianum* commercial
SMCSO: *Silybum marianum* commercial seed oil
SMSO: *Silybum marianum* seed oil
TRE: Trolox equivalent
UFA: Unsaturated Fatty Acid
24S-OHC: 24S-hydroxycholesterol
7β-OHC: 7β-hydroxycholesterol
7KC: 7-ketocholesterol
