# Supplementary material for: Characterization of Silybum marianum and Silybum eburneum seed oils: Phytochemical profiles and antioxidant properties supporting important nutritional interests
Source: PLoS One. 2024 Jun 14;19(6):e0304021. doi: 10.1371/journal.pone.0304021 (PMC11178192; doi:10.1371/journal.pone.0304021)
Supplement: S1 Table — (PDF) [file pone.0304021.s001.pdf]

S1_Table. Data of fatty acids profile of *S. marianum*, *S. eburneum*, and commercial *S. marianum* seed oils (%)

**Fatty acids**	***S. marianum***			***S. eburneum***			***S. marianum* commercial (Compagnie des sens)**		
repetition	1	2	3	1	2	3	1	2	3
**SFA**									
Lauric acid (C12:0)	1.466	0.010	0.738	1.345	0.010	0.678	0.010	0.011	0.010
Myristic Acid (C14:0)	0.086	0.096	0.091	0.126	0.055	0.090	0.100	0.105	0.095
Pentadecylic acid (C15:0)	0.020	0.027	0.024	0.021	0.024	0.023	0.010	0.011	0.010
Palmitic acid (C16:0)	17.553	8.627	13.090	13.822	11.601	12.712	9.120	9.576	8.664
Margaric acid (C17:0)	0.049	0.098	0.074	0.060	0.075	0.068	0.080	0.084	0.076
Stearic acid (C18:0)	5.200	5.397	5.299	7.637	3.063	5.350	13.510	14.186	12.835
Arachidic acid (C20:0)	1.376	3.170	2.273	2.710	1.314	2.012	3.770	3.959	3.582
Heneicosanoic acid (C21:0)	0.023	0.010	0.017	0.030	0.010	0.020	0.010	0.011	0.010
Behenic acid (C22:0)	0.339	2.295	1.317	0.948	0.589	0.768	2.690	2.825	2.556
Lignoceric acid(C24:0)	0.104	0.451	0.277	0.323	0.120	0.222	0.730	0.767	0.694
**∑SFA**	26.216	20.183	23.199	27.022	16.863	21.942	30.030	31.532	28.529
**UFA**									
**MUFA**									
(Z)-7-hexadecenoic acid (C16:1 n-9)	0.019	0.022	0.020	0.016	0.000	0.008	0.070	0.074	0.067
Palmitoleic acid (C16:1 n-7)	0.077	0.093	0.085	0.063	0.093	0.078	0.010	0.011	0.010
Margaric acid (C17:1)	0.020	0.010	0.015	0.026	0.010	0.018	0.020	0.021	0.019
Oleic acid (C18:1 n-9)	6.022	27.936	16.979	8.905	17.660	13.282	24.790	26.030	23.551
Trans-vaccenic acid (C18:1 n-7)	0.963	0.813	0.888	0.897	0.722	0.809	0.720	0.756	0.684
Cis-11-eicosenoic acid (C20:1 n-9)	0.204	0.786	0.495	0.467	0.277	0.372	0.950	0.998	0.903
C24:1 n-9	0.058	0.010	0.034	0.023	0.010	0.017	0.010	0.011	0.010
**∑MUFA**	7.363	29.671	18.517	10.397	18.771	14.584	26.570	27.899	25.242
**PUFA**									

Linoleic acid (C18:2 n-6)	59.435	50.014	54.725	45.070	63.995	54.532	37.370	39.239	35.502
α-linolenic acid (C18:3n-3)	0.010	0.173	0.091	0.010	0.412	0.211	0.320	0.336	0.304
γ-linolenic acid (C18:3 n-6)	1.970	0.010	0.990	0.500	0.010	0.255	0.010	0.011	0.010
Dihomo-gamma-linolenic acid (C20:2 n-6)	0.042	0.010	0.026	0.050	0.010	0.030	0.010	0.011	0.010
**∑PUFA**	61.457	50.207	55.832	45.630	64.427	55.028	37.710	39.596	35.825
**∑UFA**	68.820	79.878	74.349	56.027	83.198	69.613	64.280	67.494	61.066
