# Supplementary material for: Characterization of Silybum marianum and Silybum eburneum seed oils: Phytochemical profiles and antioxidant properties supporting important nutritional interests
Source: PLoS One. 2024 Jun 14;19(6):e0304021. doi: 10.1371/journal.pone.0304021 (PMC11178192; doi:10.1371/journal.pone.0304021)
Supplement: S2 Table — (PDF) [file pone.0304021.s002.pdf]

S2_Table. Data of phytosterol profile of *S. marianum*, *S. eburneum*, and commercial *S. marianum* seed oils (mg/Kg of oil)

Phytosterols	*S. marianum*			*S. eburneum*			*S.marianum* commercial (Compagnie des sens)		
**Repetition**	**1**	**2**	**3**	**1**	**2**	**3**	**1**	**2**	**3**
Unknown 1	ND	ND	ND	35.843	36.918	34.768	18.375	18.926	17.824
Campesterol	195.687	201.557	189.816	556.087	572.769	539.404	266.298	274.287	258.309
Stigmasterol	345.965	356.344	335.586	269.951	278.050	261.853	301.945	311.003	292.886
Unknown 2	14.489	14.924	14.054	88.234	90.881	85.587	91.572	94.319	88.825
Δ^7 Campesterol	140.933	145.161	136.705	93.005	95.796	90.215	135.711	139.782	131.640
Sitosterol	1511.815	1557.170	1466.461	1861.892	1917.748	1806.035	1598.607	1646.565	1550.648
Δ^5 Avenasterol	64.847	66.792	62.901	178.072	183.414	172.730	63.110	65.003	61.216
β Amyrin	215.659	222.129	209.190	116.458	119.952	112.964	151.926	156.484	147.369
Unknown 3	63.056	64.948	61.164	34.342	35.373	33.312	47.828	49.263	46.393
Δ^7 Stigmasterol	1333.318	1373.318	1293.319	365.820	376.795	354.846	1167.760	1202.792	1132.727
Δ^7 Avenasterol	83.185	85.681	80.690	141.896	146.153	137.639	27.986	28.826	27.147
24-methylene cycloartenol	85.766	88.339	83.193	130.769	134.692	126.846	94.137	96.961	91.313
Epoxysitosterol	94.530	97.366	91.694	166.412	171.404	161.420	177.236	182.553	171.919
Citrostadienol	27.641	28.470	26.812	43.767	45.080	42.454	67.169	69.184	65.154
Cholesterol	323.113	332.806	313.419	646.170	665.556	626.785	560.541	577.357	543.725
**Total content**	4500.004	4635.005	4365.004	4728.720	4870.581	4586.858	4770.201	4913.307	4627.095
