# Supplementary material for: Characterization of Silybum marianum and Silybum eburneum seed oils: Phytochemical profiles and antioxidant properties supporting important nutritional interests
Source: PLoS One. 2024 Jun 14;19(6):e0304021. doi: 10.1371/journal.pone.0304021 (PMC11178192; doi:10.1371/journal.pone.0304021)
Supplement: S3 Table — (PDF) [file pone.0304021.s003.pdf]

S3_Tabel. Data of tocopherols content of *S. marianum* (SM), *S. eburneum* (SE), and *S. marianum* commercial (SMC) seed oils (mg tocopherol/Kg of oil).

	Repetition	δ-tocopherol	γ-tocopherol	α-tocopherol	Total tocopherol content
**SM**	**1**	4.333	72.129	385.017	461.479
	**2**	4.508	71.983	385.948	462.439
	**3**	4.888	79.514	431.535	515.937
**SE**	**1**	2.280	143.340	202.598	348.217
	**2**	2.300	141.846	203.117	347.263
	**3**	2.697	169.148	240.936	412.781
**SMC**	**1**	2.995	80.536	545.273	628.804
	**2**	3.132	82.799	559.129	645.060
	**3**	2.878	81.360	548.600	632.838
