# Supplementary material for: Characterization of Silybum marianum and Silybum eburneum seed oils: Phytochemical profiles and antioxidant properties supporting important nutritional interests
Source: PLoS One. 2024 Jun 14;19(6):e0304021. doi: 10.1371/journal.pone.0304021 (PMC11178192; doi:10.1371/journal.pone.0304021)
Supplement: S4 Table — (PDF) [file pone.0304021.s004.pdf]

S5_Table. Data of phenolic profile of *S. marianum*, *S.* eburneum, and *S. marianum* commercial seed oils

	S. marianum			***S. eburneum***			***S. marianum* commercial (Compagnie des sens)**		
**Colorimetric quantification**									
**Repetition**	**1**	**2**	**3**	**1**	**2**	**3**	**1**	**2**	**3**
Total phenols content (mg GAE/100g of oil)	5.929	4.973	4.017	5.212	4.141	5.022	5.209	7.535	5.768
Total flavonoids content (mg QRE/100g of oil)	22.612	19.133	20.873	15.049	18.131	15.500	20.022	18.048	20.586
Total carotenoids content (mg/kg)	0.426	0.174	0.155	0.185	0.185	0.204	0.215	0.200	0.264
**HPLC quantification**									
**Phenols acids** (mg equivalent Quercetin / 100g of oil)									
Vanillin	0.13			0.28			0.06		
Coumarin	0.43			ND			ND		
Silibinine	ND			ND			0.36		
**Peak at 48.1 min**	3.27			ND			ND		
**Peak at 48.9 min**	5.48			ND			ND		
**Sum peak at à 280 nm**	11.81			1.94			2.54		
