# Supplementary material for: Characterization of Silybum marianum and Silybum eburneum seed oils: Phytochemical profiles and antioxidant properties supporting important nutritional interests
Source: PLoS One. 2024 Jun 14;19(6):e0304021. doi: 10.1371/journal.pone.0304021 (PMC11178192; doi:10.1371/journal.pone.0304021)
Supplement: S5 Table — (PDF) [file pone.0304021.s005.pdf]

S5_Table. Data of Antioxidant activities of *S. marianum*, *S. eburneum*, and *S. marianum* commercial seed oils

	repetition	Total antioxidant activity (mg GAE/ 100 g of oil)	DPPH (mg TRE / 100 g of oil)	FRAP (mg TRE / 100 g of oil)	KRL test	
					mg TRE / g of oil	mg GAE/ g of oil
S. marianum	**1**	26.901	7.021	0.678	252.877	101.620
	**2**	22.654	6.120	0.533	210.198	84.470
	**3**	19.114	6.645	0.557	250.283	100.000
S. eburneum	**1**	21.286	5.832	0.660	124.548	50.050
	**2**	12.772	6.171	1.415	115.675	46.485
	**3**	17.029	6.924	0.346	171.389	68.478
S. marianum commercial (Compagnie des sens)	**1**	16.527	1.315	0.177	174.776	72.247
	**2**	18.180	1.844	0.189	116.020	54.391
	**3**	17.904	2.867	0.153	139.622	60.736
