# Supplementary material for: Characterization of Silybum marianum and Silybum eburneum seed oils: Phytochemical profiles and antioxidant properties supporting important nutritional interests
Source: PLoS One. 2024 Jun 14;19(6):e0304021. doi: 10.1371/journal.pone.0304021 (PMC11178192; doi:10.1371/journal.pone.0304021)
Supplement: S6 Table — (PDF) [file pone.0304021.s006.pdf]

S6_Table: Data of valuation of the cytotoxic effects of *Silybum* seed oils (SMSO, SESO, and SMCSO) and oxysterols (7KC,7β-OHC) on THP-1 cells with the FDA test after 24 hours of treatment.

DMSO

Repetition	Control	0.041%	0.083%	0.166%	0.33%	0.66%	1.33%
**1**	99.965	101.242	99.195	97.793	91.917	89.423	89.391
**2**	100.619	100.531	100.701	98.300	94.460	89.805	88.763
**3**	99.416	102.497	101.622	100.294	90.450	89.867	88.494

Ethanol

Repetition	Control	0.0325%	0.065%	0.130%	0.260%	0.520%	1.04%
**1**	104.327	107.300	97.795	98.071	91.717	95.961	92.444
**2**	96.620	97.714	98.676	99.285	90.857	90.487	84.937
**3**	99.053	104.224	105.739	96.990	90.816	89.599	93.055

7KC

Repetition	Control	15.625 (µM)	31.25 (µM)	62.5(µM)	125 (µM)	250 (µM)	500 (µM)
**1**	96.748	94.883	79.710	67.951	49.755	42.252	34.427
**2**	99.908	77.072	76.717	65.690	50.244	43.193	36.822
**3**	103.344	91.615	66.990	55.670	42.545	43.5146	34.945

7β-OH

Repetition	Control	15.625 (µM)	31.25 (µM)	62.5(µM)	125 (µM)	250 (µM)	500 (µM)
**1**	97.962	100.694	83.502	50.748	39.819	33.953	24.062
**2**	105.269	100.576	84.443	51.549	38.732	33.783	21.652
**3**	96.769	100.433	87.282	50.632	36.206	35.505	22.110

SMSO

Repetition	Control	25 (µg/mL)	50 (µg/mL)	100 (µg/mL)	200 (µg/mL)	400 (µg/mL)	800 (µg/mL)
**1**	99.895	119.879	115.720	112.537	108.449	108.106	82.935
**2**	96.441	116.382	107.805	113.519	112.821	107.343	80.081
**3**	103.664	115.912	108.062	113.670	105.154	107.337	83.361

SESO

Repetition	Control	25 (µg/mL)	50 (µg/mL)	100 (µg/mL)	200 (µg/mL)	400 (µg/mL)	800 (µg/mL)
**1**	99.882	114.024	102.853	102.232	94.358	84.361	65.041
**2**	100.972	108.447	107.142	110.021	93.000	83.116	60.444
**3**	99.145	108.564	107.512	97.208	92.016	81.529	62.346

SMCSO

Repetition	Control	25 (µg/mL)	50 (µg/mL)	100 (µg/mL)	200 (µg/mL)	400 (µg/mL)	800 (µg/mL)
**1**	98.660	99.720	100.948	98.415	95.897	90.227	86.702
**2**	98.172	101.281	104.855	97.955	92.133	89.839	88.266
**3**	103.167	108.719	98.940	105.607	96.142	87.218	86.646
