# Supplementary material for: Characterization of Silybum marianum and Silybum eburneum seed oils: Phytochemical profiles and antioxidant properties supporting important nutritional interests
Source: PLoS One. 2024 Jun 14;19(6):e0304021. doi: 10.1371/journal.pone.0304021 (PMC11178192; doi:10.1371/journal.pone.0304021)
Supplement: S7 Table — (PDF) [file pone.0304021.s007.pdf]

S7_Table. Data of effect of *S. marianum*, *S.* eburneum, and *S. marianum* commercial seed oils on oxysterols (7-cetocholesterol and 7β-hydroxycholesterol) induced ROS overproduction in THP-1 cells.

	**DHE positive cells (%)**		
**Repetition**	**1**	**2**	**3**
**Control**	20.04	22.32	23.42
**DMSO (0.166%)**	25.29	20.14	22.61
**Eth (0.104%)**	22.57	21.35	22.41
**DMSO (0.166%) +Eth (0.104%)**	20.14	23.60	21.87
**α -tocopherol(400µM)**	23.82	21.20	27.02
**SMSO (100µg/ml)**	21.86	26.81	24.34
**SESO (100µg/ml)**	19.84	26.54	23.19
**SMCSO (100µg/ml)**	19.53	26.28	22.91
**7KC (62.5 µM)**	33.58	35.02	34.30
**7KC+ α-tocopherol (400µM)**	24.74	26.67	22.81
**7KC+ SMSO (100µg/ml)**	26.58	28.65	24.51
**7KC+ SESO (100µg/ml)**	27.69	29.85	25.53
**7KC+SMCSO (100µg/ml)**	23.88	25.74	22.02
**7β-OHC (62.5 µM)**	42.90	46.00	44.45
**7β-OHC + α-tocopherol (400µM)**	32.10	34.60	29.60
**7β-OHC + SMSO (100µg/ml**	27.20	29.32	25.08
**7β-OHC + SESO (100µg/ml)**	25.80	27.81	23.79
**7b-OHC + SMCSO (100µg/ml)**	28.30	30.51	26.09
