# Supplementary material for: Characterization of Silybum marianum and Silybum eburneum seed oils: Phytochemical profiles and antioxidant properties supporting important nutritional interests
Source: PLoS One. 2024 Jun 14;19(6):e0304021. doi: 10.1371/journal.pone.0304021 (PMC11178192; doi:10.1371/journal.pone.0304021)
Supplement: S8 Table — (PDF) [file pone.0304021.s008.pdf]

S8_Table. Data of Principal Component Analysis (PCA) (A) and Heatmap (B) of lipids profiles (fatty acids, phytosterols, and tocopherols), phenolic profiles (total phenols, flavonoids, and carotenoids content) and antioxidant activities (DPPH, FRAP, TOA, and KRL) of the three oils.

	SESO	SMSO	SMCSO
**TPC**	4.792	4.973	6.171
**Flav**	16.226	20.873	19.552
**Cart**	0.191	0.252	0.226
**TOA**	17.029	22.890	17.537
**DPPH**	6.309	6.595	2.009
**FRAP**	0.807	0.589	0.173
**KRL(T)**	137.204	237.786	143.473
**KRL(G)**	55.004	95.363	62.458
**δ-toco**	2.425	4.577	3.002
**γ-toco**	151.445	74.542	81.565
**α-toco**	215.550	400.833	551.001
**∑Toco**	369.420	479.952	635.568
**C16:0**	12.712	13.090	9.120
**C18:0**	5.350	5.299	13.510
**∑SFA**	21.942	23.199	30.030
**C18:1 n-9**	13.282	16.979	24.790
**∑MUFA**	14.584	18.517	26.570
**C18:2 n-6**	54.532	54.725	37.370
**∑PUFA**	55.028	55.832	37.710
**∑UFA**	69.613	74.349	64.280
**Campesterol**	556.087	195.687	266.298
**Stigmasterol**	269.951	345.965	301.945
**Δ7 campesterol**	93.005	140.933	135.711
**Sitosterol**	1861.892	1511.815	1598.607
**b-amyrin**	116.458	215.659	151.926
**Δ7 stigmastenol**	365.820	1333.318	1167.760
**Cholesterol**	646.170	323.113	560.541
**∑phyto**	4728.720	4500.004	4770.201
